# Molecular simulation-aided self-adjuvanting nanoamplifier for cancer photoimmunotherapy

**DOI:** 10.7150/thno.102653

**Published:** 2025-01-20

**Authors:** Junmou Gu, Rongtao Zhu, Ruopeng Liang, Weijie Wang, Jian Li, Senfeng Zhao, Yahui Wu, Jiahui Cao, Shihua Yang, Yuling Sun

**Affiliations:** 1 Department of Hepatobiliary and Pancreatic Surgery, The First Affiliated Hospital of Zhengzhou University, Zhengzhou, China.; 2 Department of Breast Surgery, Cancer Hospital of China Medical University, Liaoning, China.; 3 Institute of Hepatobiliary and Pancreatic Diseases, Zhengzhou University, Zhengzhou, 450052, China.; 4 Zhengzhou Basic and Clinical Key Laboratory of Hepatopancreatobiliary Diseases, Zhengzhou, 450052, China.

**Keywords:** drug delivery, photodynamic therapy (PDT), computational simulations, self-adjuvanting, cancer photoimmunotherapy

## Abstract

**Background**: By leveraging the power of photodynamic therapy (PDT), photosensitizers eliminate cancer cells under specific laser irradiation and trigger systemic immune responses, a potent strategy in tumor immunotherapy. However, the accompanying tumor microenvironment induces immunosuppression, restricting the efficacy of PDT.

**Methods**: Computer-aided screening was used to identify adjuvants, followed by flow cytometry analysis to assess the activation levels of various immune cells in both *in vitro* and *in vivo* experiments. Cytotoxicity assays were conducted to evaluate the impact of the nanomaterials on tumor cells. Pharmacokinetic studies were performed to observe the drug concentration *in vivo*. The efficacy of the nanomaterials was further tested using *in situ* tumor, metastasis, and organoid models.

**Results**: we first utilized computational simulations and experimental validations to identify Vit K2 as an adjuvant with a high affinity for Toll-like receptor (TLR) agonist ligands; Vit K2 effectively activated antigen-presenting cells, including dendritic cells (DCs) and macrophages, and promoted their maturation through the TLR pathways. The precisely engineered nanoamplifier with on-demand pyropheophorbide a (PPA) release ability could notably kill the primary tumor. Vit K2-activated macrophages and DCs matured, promoting antigen presentation and CD8^+^ T cell activation. As anticipated, the nanoamplifier exhibited significant anti-tumor effects in primary/distal breast tumors, lung metastatic breast cancer, and patient-derived organoid models.

**Conclusion**: Our research findings demonstrate that the nanoparticles formed by the computer-aided screened adjuvant, Vit K2, and the photosensitizer PPA achieved significant anti-tumor activity and immune microenvironment remodeling.

## Introduction

Cancer, one of the leading causes of death worldwide, severely threatens human health and results in immeasurable financial losses 1-3. Due to its insidious nature, most cancer cases are discovered during routine check-ups, significantly limiting the available treatment options 4,5. Recently, cancer immunotherapy is playing an increasingly significant role in treating advanced-stage cancer patients 6,7. Many immunotherapies, typified by checkpoint blockade agents, have been approved 8-11. For example, ipilimumab, the first FDA-approved monoclonal antibody targeting cytotoxic T lymphocytes, has shown significant efficacy 12-16. Although immunotherapy has brought hope to many patients, its specificity remains challenging, as many cancers are still unresponsive to immune checkpoint inhibitors 17-19.

Photosensitizers can generate a large amount of reactive oxygen species (ROS) under specific light conditions, leading to cell destruction 20. Localized laser irradiation can activate photosensitizers within tumor cells, facilitating photodynamic therapy (PDT) 21,22. It is noteworthy that, besides directly destroying cancer cells, PDT can induce various anti-tumor immune responses in the body, distinct from immune checkpoint inhibitors 23. After PDT, the translocation of calreticulin is facilitated from the endoplasmic reticulum to the cell membrane of cancer cells 24. The maturation of cytotoxic T lymphocytes (CTLs) and the homing of dendritic cells (DCs) are subsequently promoted 25-27. However, the immunosuppressive tumor microenvironment poses a significant obstacle to the activation and anti-tumor functionality of immune cells, a critical factor impeding the PDT therapeutic efficacy. Immunoadjuvants, substances endowed with the capacity to potentiate antigen-specific immune responses, have garnered substantial applications in vaccine development and immunotherapeutic interventions across various pathological conditions 28-29. Notably, the co-delivery of photosensitizers and immunoadjuvants, which facilitates the concomitant release of immunoadjuvants while inducing immunogenic cell death (ICD) via PDT, has emerged as a promising novel strategy for synergistic tumor immunotherapy 30.

Toll-like receptors (TLRs) are crucial pattern-recognition receptors 31. They identify foreign pathogens and are essential in the body's immune and inflammatory responses 31-32. For instance, TLR1/2 facilitates macrophage recognition of Lactobacillus jensenii via STAT3 activation 33, whereas TLR2/6 regulates myeloma onset and progression by activating macrophages 34. Due to the remarkable characteristics of TLRs, an increasing number of vaccine adjuvants related to TLR ligands are being applied in clinical settings.

**Figure 1:**
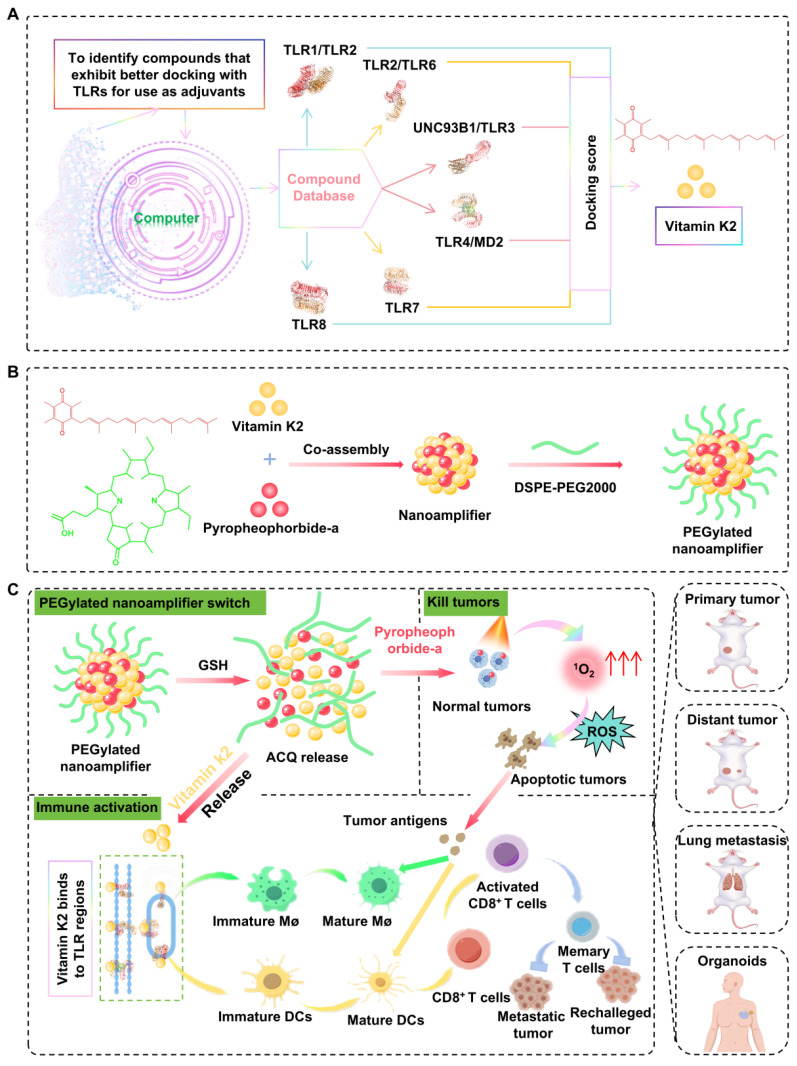
** Schematic diagram of molecular simulation-aided self-adjuvanting nanoamplifier for cancer photoimmunotherapy.** The nanoamplifier was constructed through the supramolecular co-assembly of the computer-aided screened adjuvant Vit K2, which binds to TLRs and the photosensitizer PPA. This photo-immunotherapy combines photodynamic therapy with immunotherapy, initiating the activation of macrophages and DCs and culminating in the induction of T-cell differentiation. In multiple tumor-bearing and organoid models, it demonstrated powerful tumor-eliminating and immune-activating capabilities.

Herein, to fully harness the potential of photosensitizers in tumor treatment while addressing their limitations in treating deep-seated tumors and enhancing immune effects, we reported a molecular simulation-aided self-adjuvanting nanoamplifier for cancer photoimmunotherapy. The computational simulations and experimental validations were employed to identify a TLR-related small molecule, Vitamin K2 (Vit K2), as an adjuvant. The precisely engineered nanoamplifier integrated molecular simulation-screened Vit K2 with the photosensitizer, pyropheophorbide-a (PPA). The addition of a small amount of 1,2-distearoyl-sn-glycero-3-phosphoethanolamine-N-methoxy-(polyethylene glycol) 2000 (DSPE-PEG2000) was incorporated into nanoamplifier, bestowed upon the nanoamplifier excellent hydrophilicity and stability. As expected, the PEGylated nanoamplifier demonstrated long *in vivo* circulation time, favorable intratumoral biodistribution, direct oncolytic effect under specific irradiation, DC and macrophage maturation, and cytotoxic T cell infiltration, resulting in potent photoimmunotherapeutic efficacy in multiple mouse models and a patient-derived organoid model (Figure 1).

## Materials and Methods

### Cell lines and animals

4T1, Hep1-6, DC 2.4, B16, and RAW 264.7 cell lines were gifts from Shenyang Pharmaceutical University. 4T1 and DC 2.4 cell lines were cultured in a complete medium consisting of 89% DMEM, 10% fetal bovine serum, and 1% penicillin-streptomycin (100×). Hep1-6 and RAW 26.7 cell lines were cultured in a complete medium consisting of 89% RPMI-1640, 10% fetal bovine serum, and 1% penicillin-streptomycin (100×). All cell lines did not show any signs of mycoplasma infection and were cultured in an incubator at 37 °C, 5% CO_2_ and 85% to 90% humidity, passaged using trypsin digestion, and cryopreserved in the serum-free cryopreservation medium.

Female BALB/c mice were supplied by Huafukang Bioscience (Beijing, China) and housed at Shenyang Pharmaceutical University (Shenyang, China). The mice were acclimatized in the housing facility for one week before starting the animal experiments. The methods of animal euthanasia involved either a natural death or euthanasia by cervical dislocation following deep anesthesia. All experimental procedures were approved by the Institutional Animal Care and Use Committee of Shenyang Phaemaceutical University.

### Molecular simulations and screening

First, the Traditional Chinese Medicine (TCM) compound was established from the database by locating the "substance" module under "subsets" in Zinc (https//zinc.docking.org/substances/subsets/tcm/). The TCM library contains a total of 35161 compounds with an option for bulk download in SDF format. The pH range was set to 7.0 ± 2.0 to generate all possible ionization states. The generation of chiral isomers was avoided. After processing, TCM library containing 53,115 molecules was obtained and saved as the finalized Maestro-format TCM library. All other parameters were kept at their default settings. TLR target-related proteins were searched by navigating to the Protein Data Bank (PDB) website at https//www.rcsb.org/ and searching for TLR. Target proteins, such as TLR8, TLR7, TLR4/MD2, UNC93B1/TLR3, TLR2/TLR6, or TLR1/TLR2 with lower resolution values more suitable for virtual screening purposes, were selected from the search results. Finally, the protein structures were downloaded as a PDB file.

Linux terminal was opened, and Maestro was typed to launch Maestro 9.7. Subsequently, Protein Prepare was entered in the search bar to launch the search. From the results, the Protein Prepare tool was selected to open the interface. The desired protein PDB file was downloaded in the Import window, leaving all other parameters at their default settings.

Open Maestro 9.7 (as described previously). In the tool search bar at the top right, type Receptor Grid Generation and press Search. Select Receptor Grid Generation to access the grid generation interface. In the grid position selection, choose the mode Determine position based on ligand. Check the option to View Grid to preview the docking grid being generated. Click on Ligand to define the position, then select the prepared protein's ligand (e.g., NOG) as the reference for grid generation. Keep all other parameters at their default settings. Click Run in the bottom right corner and wait for the process to be completed. Once finished, you will have a docking grid generated based on the protein's ligand position.

Open the Ligand Docking tool in Maestro (as described previously). In the Ligand Docking interface, locate the Grid File input field at the top. Import the docking grid file by selecting the protein grid file you just generated (in ZIP format). To perform ligand docking using the prepared TCM drug library with multiple target proteins. Open Maestro and search for the Ligand Docking tool in the top-right search bar (similar to Step 4). Open the ligand docking interface. At the top of the docking interface, locate the Grid File Input section. Import the grid file generated in the earlier steps (in ZIP format) for the specific target protein. In the Ligand Input section, choose Files as the input method. Import the Maestro-format TCM drug library prepared earlier. Keep the default docking precision as Standard Precision (SP). Do not modify other parameters unless required. Click Run in the bottom-right corner to initiate docking. Wait for the docking process to be completed. This will generate docking results, including binding poses and scores, for each compound in the TCM library against the specified protein. Evaluate the docking results based on interaction energies, steric hindrance, and binding modes. The different binding modes were scored to obtain docking scores. A lower docking score indicates a more stable binding. Perform the docking process for each of the six target proteins include TLR8, TLR7, TLR4MD2, UNC93B1/TLR3, TLR2/TLR6, and TLR1/TLR2, using their respective grid files. Note that some molecules may not dock into the active site of certain proteins and will not produce a docking pose or score. Identify molecules that successfully dock with the active sites of all six target proteins. Sum the docking scores for each molecule across the six proteins to calculate a composite score. Rank the molecules in ascending order of their composite scores to identify the top candidates for further analysis. A total of 30,482 molecules that can bind to the active sites of all six target proteins are identified (Table S1). The composite scores for these molecules are calculated, and the molecules are ranked by their combined scores to highlight the most promising candidates.

### Real-time quantitative PCR (RT-qPCR)

Vit K2, Vit B1, and Scutellarin were dissolved in DMSO. The log-phase DC2.4 cells were divided into 0.1% DMSO, 3 experimental groups, each with 0.1 μmol/L of Vit K2, Vit B1, or Scutellarin. RNA was extracted employing the Total RNA Isolation Kit. Subsequently, the RNA concentration was measured using Nanodrop one (Thermo, America). RNA was then reverse transcribed into cDNA. Finally, the cDNA was extended into DNA using the ChamQ Blue Universal SYBR qPCR Master Mix and real-time quantitative analysis was performed using the CFX96™ (Bio-rad, America). The primers used in the experiments are shown in Table S2.

### Western blotting

The DCs were divided into control group, the Vit K2 group and the Pam3CSK4 group. No additional drugs were added in the control group. For the Vit K2 and Pam3CSK4 groups, Vit K2 and Pam3CSK4 were added a final concentration of 0.1 μmol/L. After 12 h, proteins were extracted from the cells. Equal amounts of protein were loaded into the wells of a 10% precast gel. The transfer time for different target proteins varied, with all conditions set at 300 mA. The PAGE membranes were blocked with a rapid blocking solution. The blocking solution was then removed, and wash the membranes. The membranes were incubated with primary antibodies at 4°C for 16 h. Subsequently, wash the membranes and add diluted secondary antibodies. Then, wash the membranes. The membranes were exposed using the ECL system.

### Flow cytometry

The flow cytometry antibodies for all mouse-derived cells are as follows: CD45 (Biolegend 103129), CD3 (Biolegend 100203), CD8a (Biolegend 100711), CD11c (Biolegend 117309), FOXP3 (Biolegend 126403), CD44 (Biolegend 103007), CD80 (Biolegend 104705), CD25 (Biolegend 102011), CD62L (Biolegend 104427), CD86 (Biolegend 105105), CD4 (Biolegend 100469). Flow cytometry data were collected manipulating (Becton, Dickinson and Company) and processed with FlowJo software.

### Analysis of maturation results for DC2.4 and RAW 264.7 cells

Four different complete media were added to DC2.4 cells or RAW 264.7 cells at a logarithmic phase of 1×10^5^ cells. One group was treated with 10 μL of DMSO as a blank control, while the other three groups contained complete media with a final concentration of 0.1 μmol/L Vit K2, Vit B1 or Scutellarin. After 12 h, the maturation level of DC2.4 cells was measured using CD80 and CD86 antibodies and that of RAW 264.7 cells was determined based on CD80 and F4/80 antibodies.

### Synthesis of PEGylated nanoamplifier

PPA and Vit K2 were dissolved at 3 µmol/mL, and DSPE-PEG2000 was dissolved at 1 mg/mL in tetrahydrofuran. Then, 30 µL of the PPA solution, 60 µL of the Vit K2 solution, 19 µL of the DSPE-PEG2000 solution and 91 µL of tetrahydrofuran solvent were mixed. Next, the mixture was added dropwise to a vial containing 1 mL of ddH_2_O. After stirring for 8 min, the mixture was transferred to a rotary evaporator to remove the tetrahydrofuran, resulting in the desired PEGylated nanoamplifier.

### Characteristics of PEGylated nanoamplifier

The particle size and zeta potential of the PEGylated nanoamplifier were measured using the specified volumes and data were obtained using the Nano Zetasizer (UK). The morphology and size of the PEGylated nanoamplifier were analyzed by the electron microscope (JEOL 100CX II, Japan). To confirm the chemical composition and structure, Fourier transform infrared (FTIR) was used to analyze the spectra of PPA, Vit K2, and PEGylated nanoamplifier ranging from 400 to 4000 cm⁻¹ at room temperature.

### Stability of nanoparticles

PEGylated nanoamplifieres were stored at 4°C for one week, and their particle size were measured at 4 time points (0, 2, 4, 6 day and compared under the same conditions. In addition, 1 mL of PEGylated nanoamplifier solution was placed in 19 mL of various concentrations (10, 20, 40, 60, 80 mM) of SDS, NaCl, Urea, or Na_2_S_2_O_4_ solutions. After 2 h at 37 °C for , the particle sizes of PEGylated nanoamplifiers in the mixture were measured. Then, PEGylated nanoamplifier was added to different concentrations of GSH. After 12 h at 37 °C the release rate of the nanoparticles was measured at 5 time points.

### Release experiment

The PEGylated nanoamplifier solution containing 1 mg of PPA in 1 mL was placed into a dialysis bag immersed in 30 mL of PBS containing different concentrations of GSH. The samples were incubated in a shaker at 37 ℃ and 100 rpm. At 0, 2, 4, 8, and 12 h, the absorbance at 670 nm wavelength was measured to calculate the release amount of PPA.

### Aggregation-caused quenching relief

DTT was added to PEGylated nanoamplifiers containing an equal concentration of 5 µmol/L PPA to achieve a final concentration of 2 mM. The mixtures were incubated at 37 °C for 6 h. Subsequently, their fluorescence spectra were recorded at 0, 2, 4, and 6 h using a full-wavelength microplate reader at 415 nm excitation and 675 nm emission.

### ^1^O_2_ detection

GSH was added to the PEGylated nanoamplifier solution containing an equal concentration of 5 µmol/L PPA to achieve a final concentration of 1 mM. At the 0, 2, and 4 h time points, add SOSG probe. Then, the mixtures were irradiated for 5 min using a 660 nm laser at 200 mW cm^-2^. Finally, the fluorescence intensity of the mixtures was measured using a microplate reader at 504 nm excitation and 525 nm emission.

### Cellular uptake

1×10^5^ 4T1 cells were cultured in a 12-well plate. Once fully adhered, 1.5 ml of complete medium containing PEGylated nanoamplifier (PPA concentration of 1 µg/mL) was added. Then, DAPI was added to the cells at 2, 4, and 6 h time points. Finally, photographs were taken using a confocal microscope.

### Observation of intracellular ROS

1×10^5^ 4T1 cells were cultured in a 12-well plate. Once the cell concentration reached 60%, divided into five groups (PBS, PPA, PEGylated nanoamplifier, PPA+L, PEGylated nanoamplifier+L). For the PBS group, PBS was added to complete medium. For the remaining 4 groups, PPA or PEGylated nanoamplifier was added to complete medium (PPA concentration of 1 µg/mL). Importantly, 6 h after adding the reagents, the cells were incubated with an appropriate concentration of DCFH-DA reagent for 30 min. Then, the PPA+L and PEGylated nanoamplifier+L groups were irradiated with a 660 nm laser at 200 mW cm^-2^ for 5 min. Finally, confocal microscopy was used to observe the fluorescence intensity of intracellular ROS production.

### Cytotoxicity assay

3×10^3^-5×10^3^ 4T1 or Hep1-6 cells were added to 96-well plates. Cells were divided into 6 groups (Control, PBS, PPA, PEGylated nanoamplifier, PPA+L and PEGylated nanoamplifier+L) with five replicates per group. First, 10 mL RPMI-1640 medium was added to the three 15 mL sterile enzyme-free centrifuge tubes. Then 300 µL PBS was added to one tube and an appropriate amount of PPA or PEGylated nanoamplifier to the other two tubes (PPA concentration of 1 µg/mL). The cells were washed twice with PBS. A blank control group containing only 100 µL medium was set up without cells. The control group contained 100 µL medium with cells, and the PBS group contained 100 µL medium with PBS. The PPA and PPA+L groups contained 100 µL of medium with PPA. The PEGylated nanoamplifier and PEGylated nanoamplifier+L groups contained 100 µL of medium with PEGylated nanoamplifier. After 12 h, the PPA+L and PEGylated nanoamplifier+L groups were irradiated. After another 24 or 48 h, 10 µL of the 3-(4,5-Dimethylthiazol-2-yl)-2,5-diphenyltetrazolium bromide (MTT) solution was added. After 4 h, DMSO was added, and shake the plate for 10 min. Finally, evaluate the absorbance at 570 nm. The cell viability was calculated using the formula: cell survival rate = (experimental group - blank control) / (control group - blank control) × 100%.

### Observation of cell morphology under confocal microscopy

The treated 4T1 and Hep1-6 cells were stained with Calcein AM/PI detection working solution. Stain the DC or Macrophage cells with an appropriate concentration of DiIC18 (3) (Beyotime, China). The cells are then washed 1-2 times with PBS, followed by staining the nuclei with DAPI. Finally, the changes in cell morphology were observed under confocal microscopy, where blue indicated the nuclei and red represented the cell membrane.

### Immune activation effects of PEGylated nanoamplifier *in vitro*

4T1 cells (3×10^3^-5×10^3^) were added to the upper chamber. The experiment was divided into three groups: PBS, PPA+L, and PEGylated nanoamplifier+L and respective formulations were applied. After 12 h, the treated 4T1 cells were transferred from the upper chamber to the lower chamber containing DCs or macrophages. After an additional 12 h, the polarization state of the macrophages was analyzed by flow cytometry. T cells were then added to the lower chamber of the DC-containing Transwell plate and co-cultured for 48 h. Subsequently, the differentiation of T cells and maturity of DC were assessed by flow cytometry. The extraction of T cells was carried out according to the instructions provided in the mouse CD3^+^ selection kit (B90021, Selleck, China).

### Pharmacokinetics study *in vivo*

PPA or PEGylated nanoamplifier solutions were administered intravenously via the tail vein into BALB/c mice at 5 mg/kg of PPA. Live imaging (IVIS Lumina Series 816 III) was used to photograph BALB/c mice at 8, 12, and 24 h intervals. At 24 h, the mice were dissected to observe the PPA content in the heart, liver, spleen, kidneys, and lungs using a live imaging system.

### Inhibitory effect of the formulations on *in situ* tumors

36 mice were housed in six differently labeled cages: PBS, PPA, PEGylated nanoamplifier, PPA+L, P@P+L, and PEGylated nanoamplifier+L groups. P@P refers to nanoparticles formed by PPA and 15% DSPE-PEG2000. First, 3×10^6^ 4T1 cells were injected into the second mammary pad on the right side of each mouse to establish an *in situ* tumor model. Ten days after the tumor cell inoculation, 100 µL of PBS, PPA, P@P, or PEGylated nanoamplifier formulation (the latter five groups receive a PPA dose of 5 mg/kg) were administered via tail vein injections. The PPA+L, P@P+L, and PEGylated nanoamplifier+L groups were irradiated at the tumor site 12 h post-administration. Treatments were repeated every two days and tumor volume and mouse weight were recorded on days 10, 12, 14, 16, and 18. Tumors were excised weighed, and photographed.

Flow cytometry was used to analyze the proportions of immune cells in the blood and tumors. Additionally, H&E staining was performed on sections of the mice's hearts, livers, spleens, kidneys, and lungs.

### Establishment of a bilateral tumor model

Twenty BALB/c mice were housed in four differently labeled cages: PBS, PEGylated nanoamplifier, PPA+L, and PEGylated nanoamplifier+L groups. Initially, 3×10^6^ 4T1 cells were injected into the second mammary pad on the right side of each mouse to establish an *in situ* tumor model. Seven days after tumor cell inoculation, 100 µL of PBS, P@P formulation, or PEGylated nanoamplifier formulation (the latter three groups receiving a PPA dose of 5 mg/kg) via tail vein. The PPA+L group and PEGylated nanoamplifier+L group were irradiated on the tumor site 12 h post-administration. Treatments were repeated every two days for a total of four times. After the fourth treatment, the therapy was stopped. On day 15 post-tumor inoculation, and 3×10^6^ 4T1 cells were injected into the left hip. On day 19, distal tumors were photographed every 3 days for a total of 4 times. On day 29, mice were euthanized, proximal and distal tumors and lung tissues were extracted and photographed.

### Patient-derived organoids model

Organoids from a patient's breast cancer tumor were obtained following the instructions provided in the Organotial Human Breast Cancer Organoid Culture Kit (Shanghai, China). After 7 days, different formulations were added to the organoids, which were irradiated 12 h later. Following the kit instructions, immune cells were isolated from the patient's axillary lymph nodes and co-cultured with the organoids. The maturity of DCs was measured 12 h after co-culturing by flow cytometry, T cell differentiation was assessed 72 h later.

### Statistical analysis

The data were analyzed using GraphPad Prism 8 software. Statistical significance was indicated by *p < 0.05, **p < 0.01 and ***p < 0.001, and 'ns' denoted not significant.

## Results and Discussion

### Adjuvant selection and validation

**Figure 2:**
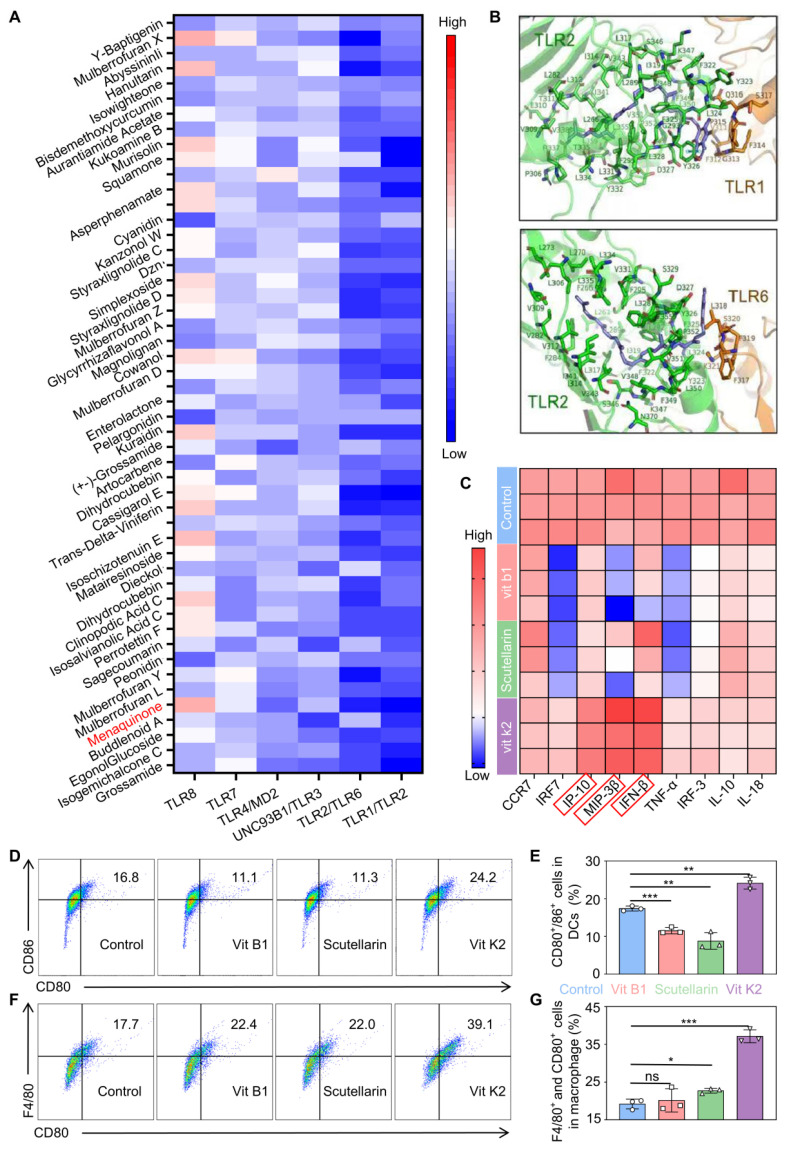
** Simulation discovery of the adjuvant and validation of its associated functions.** (A) Computer simulations for docking scores with TLRs: the top 50 small molecules (red showing high scores, blue indicating low scores). (B) Simulated illustration of Vit K2 docking with TLR1/2 and TLR2/6. (C) Transcriptional expression of Th1-type cytokines in DCs after 4 h of stimulation with Scutellarin, Vit B1, or Vit K2 (n = 3). (D) Examination of CD80^+^/86^+^ cell populations in DCs via flow cytometry following 12 h exposure to Control, Vit B1, Scutellarin or Vit K2 (n = 3). (E) Statistical evaluation of CD80^+^/86^+^ cells in DCs (n = 3). (F) Flow cytometry analysis of F4/80^+^ and CD80^+^ cells in macrophages after 12 h of stimulation with Control, Vit B1, ifScutellarin or Vit K2 (n = 3). (G) Statistical evaluation of F4/80^+^ and CD80^+^ cells in macrophages (n = 3). *P*-value: ns (no significance) *p* > 0.05, *p < 0.05, **p < 0.01, ***p < 0.001.

The compounds from TCM liberary was docked with TLR1/TLR2, TLR2/6, UNC93B1/TLR3, TLR4/MD2, TLR7, and TLR8. Some matches were scored based on suitability, as shown in Figure 2A. Vit K2 was identified as an adjuvant with a high affinity for TLR agonist ligands. Figure 2B and Figure S1 illustrate the molecular binding of Vit K2 with the above-mentioned TLRs. To verify that Vit K2 ccould activate the TLR2 pathway, we compared it with the TLR2 agonist Pam3CSK4. As shown in Figure S2A-C, the expression levels of TLR2, MyD88, and NF-κB proteins in the TLR2 pathway were higher in both the Vit K2 and Pam3CSK4 than the Control. The relative RNA expression levels of TLR2 and IP-10 significantly increased in the Vit K2 and Pam3CSK4, indicating that Vit K2 could activate the TLR2 pathway.

Moreover, to verify whether Vit K2 could activate DCs, we first assessed whether DMSO significantly influenced DC activation or RNA expression at 0.1% concentration as a solvent. As shown in Figure S3A-D, the DMSO group exhibited no discernible impact on DC activation compared to the control group, nor were there significant changes in the relative RNA expression levels of TLR2 and IP-10. Then, we divided DCs into four groups: DMSO as Control, Vit B1, Scutellarin, and Vit K2. Among them, Vit B1 and Scutellarin were the negative controls.

The results of RT-qPCR verified the differences in cytokine production within DCs. Importantly, the activation of DCs might be associated with the high expression of Th1 cytokines. An increase in Th1 cytokines could enhance antigen-presenting cells' maturation, processing, and presentation of antigens. The Vit K2 group significantly increased IP-10, MIP-3β, and IFN-β compared to the Control (Figure 2C). However, the Vit B1 and Scutellarin groups showed a decrease in specific cytokines compared to the Control. Subsequently, we validated the effects of four formulations on the activation of DCs and M1-type macrophages by flow cytometry We found the percentages of mature DCs to be 17.4 ± 0.66, 11.5 ± 0.84, 8.78 ± 2.19, and24.1 ± 1.60 in the Control, Vit B1, Scutellarin, and Vit K2 groups, respectively (Figure 2D-E). The percentages of M1-type macrophages for the Control, Vit B1, Scutellarin, and Vit K2 groups were 19.2 ± 1.31, 20.2 ± 3.09. 22.7 ± 0.64, and 37.1 ± 1.72, respectively (Figure 2F-G). Thus, the Vit K2 group had statistically significant effects on DC maturation and M1-type macrophage polarization compared to the other groups.

### Preparation and characterization of nanomaterials

We investigated whether Vit K2 could form a nanoamplifier with photosensitive PPA. We studied the optimal ratio of PPA to Vit K2 by co-assembling them in ratios of 1:5, 1:2, 1:1, 2:1, and 5:1. The results indicated that when the ratio of PPA to Vit K2 was 1:2, the nanoamplifier had the smallest diameter and the lowest PDI (Figure S4). Also, adding a small amount of DSPE-PEG2K could enhance the hydrophilicity and stability of the nanoamplifier. We added 15% DSPE-PEG2000 and measured particle sizes, which were 164.7 ± 6.86 nm (1:5), 155.2 ± 4.54 nm (1:2), 143.7 ± 3.01 nm (1:1), 175.3 ± 1.95 nm (2:1) and 196.5 ± 3.02 nm (5:1). Their PDI values were 0.144 ± 0.05 (1:5), 0.113 ± 0.04 (1:2), 0.172 ± 0.03 (1:1), 0.165 ± 0.01 (2:1) and 0.089 ± 0.03 (5:1) (Figure S5). As DSPE-PEG2000 was added, the zeta potential of the nanoamplifier shifted from a positive to a negative value, facilitating cellular uptake (Figure S6). As shown in Figure S7, the peaks at 1729 cm⁻¹, 1658 cm⁻¹, 1384 cm⁻¹, and 1297 cm⁻¹ in the formulation confirmed that PPA and Vit K2 were the basic components of the PEGylated nanoamplifier. Specifically, the peaks at 1729 cm⁻¹ and 1384 cm⁻¹ were attributed to Vit K2, while 1658 cm⁻¹ and 1297 cm⁻¹ corresponded to PPA. The characteristic peaks from both samples appeared in the formulation, validating their presence. Moreover, the emergence of new peaks and peak shifts in the formulation suggested the formation of nanoparticles through the interaction between PPA and Vit K2. Notably, the 3399 cm⁻¹ peak exhibited a red shift, likely associated with hydrogen bond formation.

**Figure 3:**
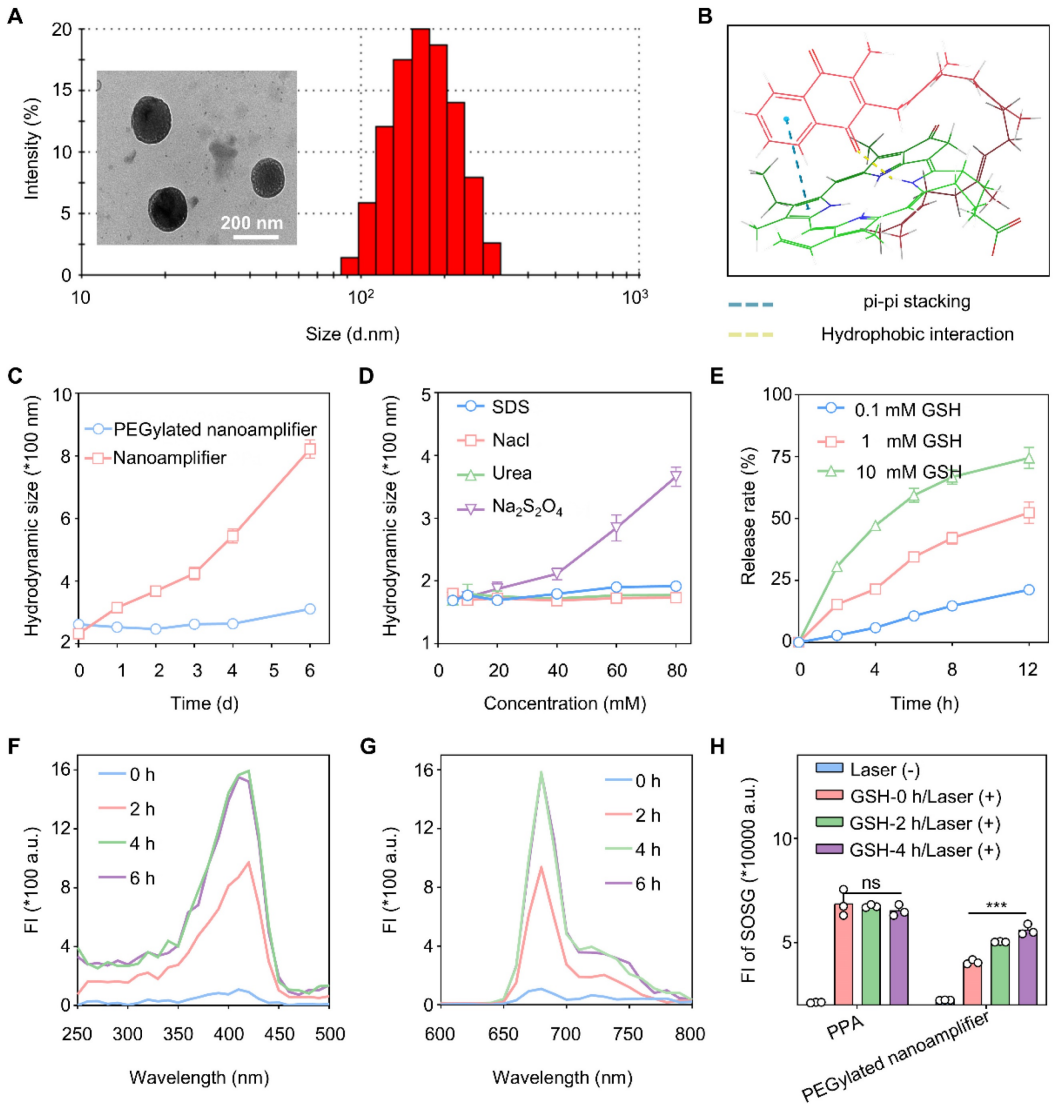
** Characterization of PEGylated nanoamplifier.** (A) TEM and size distribution in images of PEGylated nanoamplifier. (B) Intermolecular interactions between Vit K2 and PPA. (C) Analysis of PEGylated nanoamplifier NAs and nanoamplifier particle size at 0, 1, 2, 3, 4, 5, and 6 day time points (n = 3). (D) Changes in particle size of the PEGylated nanoamplifier in SDS, NaCl, Urea, or Na_2_S_2_O_4_ solutions at concentrations of 10, 20, 40, 60, or 80 mM (n = 3). (E) PPA release rate of the PEGylated nanoamplifier at different GSH concentrations over 0, 2, 4, 6, 8 and 12 h (n = 3). (F-G) Fluorescence spectra change of the PEGylated nanoamplifier at 0, 2, 4, and 6 h at 1 mM GSH concentration. (H) SOSG fluorescence signal intensity detection of PPA and PEGylated nanoamplifier at 0, 2, 4, and 6 h under a 1 mM GSH concentration with 660nm laser irradiation (n = 3). *P*-value: ns (no significance) *p* > 0.05, *p < 0.05, **p < 0.01, ***p < 0.001.

Figure 3A shows the particle size distribution and electron microscopy images of nanoparticles with a 1:2 PPA to Vit K2 ratio, including 15% DSPE-PEG2000. Over one week in water, the nanoamplifier showed significantly greater particle size variations than the PEGylated nanoamplifier. In addition, we simulated the stability of the supramolecular nanoamplifier and the results are shown in Figure 3C. We also simulated molecular co-assembly using software and annotated the potential interactions involved. The results showed that PPA and Vitamin K2 assembled through pi-pi stacking and hydrogen bonding (Figure 3B). Furthermore, Figure S8 reveals that the PEGylated nanoamplifier had a slower rate of particle size increase in 10% FBS compared to the nanoamplifier, possibly attributed to the incorporation of DSPE-PEG2000, altering the nanoparticle charge and reducing serum protein-induced degradation of the nanoparticles.

We also measured particle size of PEGylated nanoamplifier in SDS, NaCl, Urea, and Na_2_S_2_O_4_ at concentrations of 5, 10, 20, 40, 60, and 80 mM. Figure 3D shows that the PEGylated nanoamplifier had the biggest particle size variation in Na_2_S_2_O_4_, with slight changes in SDS, NaCl, and Urea solutions. These results of molecular docking and experimental validations indicated that size of PEGylated nanoamplifier was significantly affected by reducing agents. Notably, in SDS solutions ranging from 20 mM to 80 mM, the particle size of the PEGylated nanoamplifier gradually increased, indicating that hydrogen bonds and hydrophobic interactionsits significantly influenced its size.

The PEGylated nanoamplifier was added to various concentrations of GSH to observe the PPA release rate and study the release behavior in the simulated tumor environment. Figure 3E shows that as the concentration of GSH increased, the PEGylated nanoamplifier's release rate also gradually increased, demonstrating a faster release of the PEGylated nanoamplifier in the tumor microenvironment than in normal tissues. Figure 3F-G illustrates that the fluorescence intensity of the PEGylated nanoamplifier increased in a time-dependent manner over 6 h, with the enhanced fluorescence intensity attributable to the collapse of the PEGylated nanoamplifier triggered by DTT. We also used the SOSG assay kit to detect singlet oxygen production in PPA and PEGylated nanoamplifier solutions before and after the addition of DTT. Figure 3H reveals that DTT did not notably influence singlet oxygen production in the PPA solution. Conversely, the addition of GSH notably increased the singlet oxygen in the PEGylated nanoamplifier. These results suggested that the formation of a PEGylated nanoamplifier could mitigate aggregation-caused quenching (ACQ), benefiting PPA-mediated PDT and enhancing the killing of tumor cells.

### Cell uptake, *in vitro* anti-tumor effects, and immune activation

**Figure 4:**
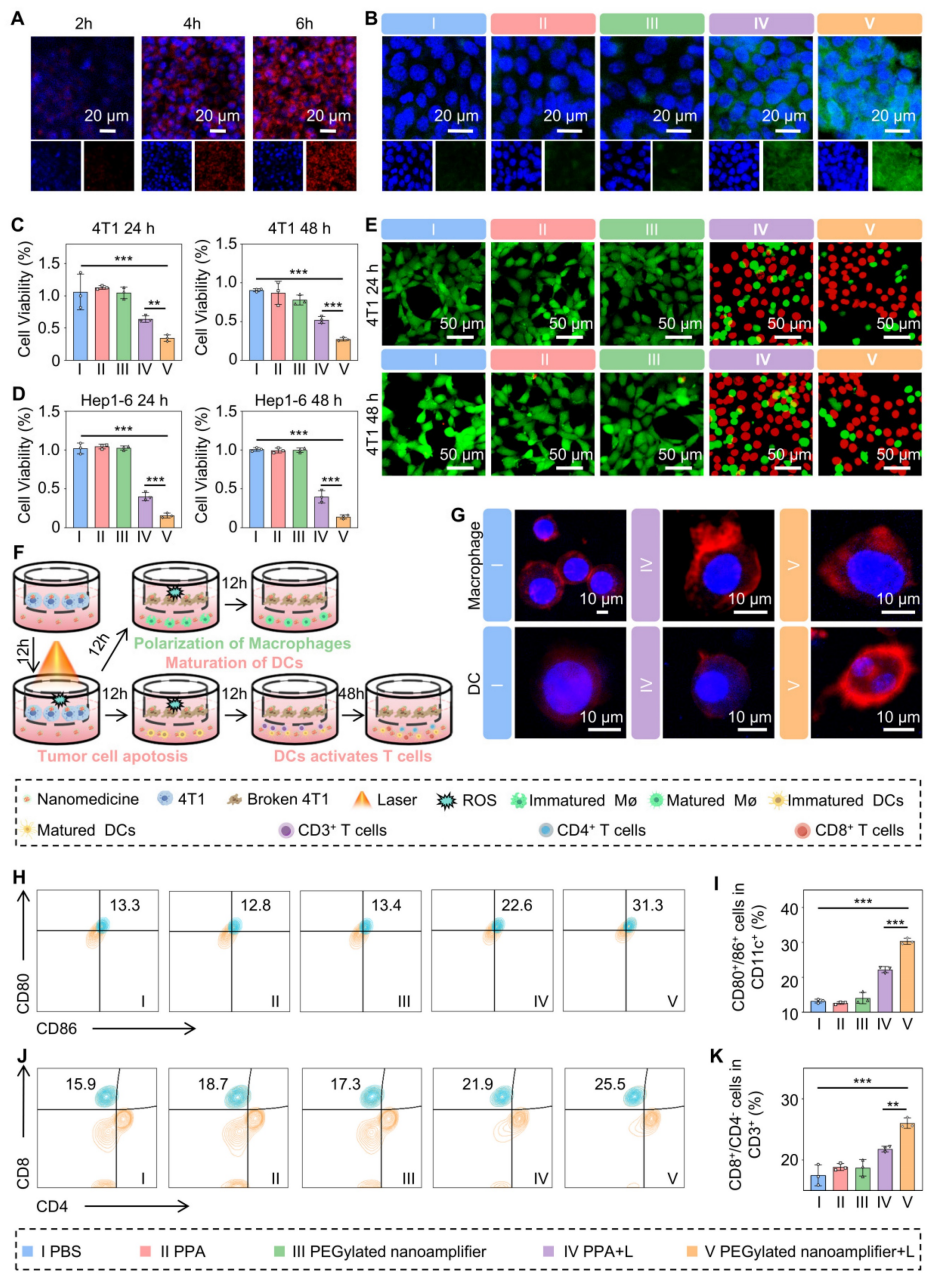
***In vitro* uptake, antitumor, and immune activation effects of PEGylated nanoamplifier.** (A) Uptake of PEGylated nanoamplifier by 4T1 cells at 2, 4, and 6 h. (B) Analysis of ROS in 4T1 cells after 6-h treatment with PBS, PPA, PEGylated nanoamplifier, PPA+L or PEGylated nanoamplifier+L groups. (C-D) Cytotoxicity analysis of Hep1-6 and 4T1 cells after 24- or 48-h treatment with PBS, PPA, PEGylated nanoamplifier, PPA+L or PEGylated nanoamplifier+L groups (n = 3). (E) Live/dead images of 4T1 cells under confocal microscopy (Green indicates healthy cells while red represents apoptotic or necrotic cells). (F) Schematic diagram of the treatment of 4T1 cells and their co-culture with immune cells. (G) Morphological changes of macrophages and DC cells observed under a confocal microscope (red represents cell membranes, blue represents cell nuclei). (H) Evalution of CD80^+^/86^+^ cells in CD11c^+^ using flow cytometry analysis (n = 3). (I) Evalution of CD80^+^/86^+^ cell stastistics in CD11c^+^ (n = 3). (J) Flow cytometry evaluation of CD8^+^/CD4^-^ cells within CD3^+^ (n = 3). (K) Evalution of CD8^+^/CD4^-^ cells stastistics in CD3^+^ (n = 3). PBS (I), PPA (II), PEGylated nanoamplifier (III), PPA+L (IV), PEGylated nanoamplifier+L (V). *P*-value: ns (no significance) *p* > 0.05, *p < 0.05, **p < 0.01, ***p < 0.001.

We utilized confocal laser scanning microscopy to observe variations in specific fluorescence intensity within the cellsto evaluate the cellular uptake efficiency of the PEGylated nanoamplifier and ROS generation. Figure 4A and Figure S9 show that the uptake of PEGylated nanoamplifier by 4T1 and Hep1-6 cells progressively increased over 6 h. Figure 4B displays the fluorescence intensity of intracellular ROS in 4T1 cells after 6 h of incubation in PBS, PPA, and the PEGylated nanoamplifier. Notably, the PEGylated nanoamplifier+L group generated significantly more ROS than the PPA+L group, likely due to the enhanced cellular uptake.

Next, we used the MTT assay to assess the photodynamic cytotoxic effects of each formulation on 4T1 and Hep1-6 cells. Figures 4C-D demonstrate the highest cytotoxicity of PEGylated nanoamplifier+L against 4T1 and Hep1-6 cells, followed by PPA+L. The remaining three groups showed no significant differences in cytotoxic effects. To further validate our findings, we used a cytotoxicity assay kit to stain the cells and observed them under a confocal microscope. In this assay, red fluorescence represented apoptotic cells, while green fluorescence indicated viable cells. The results showed that the PEGylated nanoamplifier+L group exhibited the greatest percentage proportion of red cells followed by the PPA+L group (Figure 4E, Figure S10). The other three groups exhibited no significant differences, consistent with our previous MTT assay results.

To assess whether the PEGylated nanoamplifier retained its immunostimulatory effect on DCs after modification, DMSO, PPA, the PEGylated nanoamplifier, or Vit K2 were mixed into the complete medium of DCs. In the Control group, 0.1% DMSO was added. The final concentration of PPA was 0.05 μmol/Lin the PPA, PEGylated nanoamplifier, and PPA with Vit K2 groups. The Vit K2 concentration was at 0.1 μmol/L in the PPA+Vit K2 group. The results revealed that the PPA+Vit K2 group had the highest level of DC maturation, followed by the PEGylated nanoamplifier group. In contrast, the PPA and DMSO groups displayed the lowest maturation levels (Figure S11). DC maturation in the PEGylated nanoamplifier group was not as high as in the PPA+Vit K2 group, possibly due to the lack of high concentrations of GSH in the DCs, which may have affected the release of Vit K2 in the PEGylated nanoamplifier group.

Mature DCs exhibit numerous dendrites, whereas immature DCs contain more cytoplasm than mature DCs. M2 macrophages typically have a round or oval shape, while M1 macrophages are spindle-shaped 35. Confocal microscopy validated the morphological changes in DCs and macrophages, providing direct evidence of immune activation. We placed 4T1 cells in Transwell chambers and divided them into three groups treated with PBS, PPA+L, or PEGylated nanoamplifier+L (Figure 4F). After 12 h, we transferred the Transwell chambers to culture dishes containing DCs or macrophages. Following a 12-h co-culture period, DCs developed numerous dendritic extensions on their surface, and macrophages transformed into a spindle shape, consistent with previous studies (Figure 4G).

Next, we conducted flow cytometry experiments to delve deeper into the impact of mature DCs on T-cell differentiation (Figure S12). Figure S13A-B shows no significant changes in the proportion of CD3^+^ cells within CD11c^-^ cells during co-culturing in each group. Figure 4H-K indicates that the proportion of mature DCs and CD8^+^ T cells was highest in the PEGylated nanoamplifier+L group, followed by the PPA+L group. This observation suggested that the PEGylated nanoamplifier could promote the activation of DCs, thereby increasing the number of CD8^+^ T cells.

To validate the universality and versatility of the PEGylated nanoamplifier, B16 cells were divided into five groups and treated with PBS, PPA, PEGylated nanoamplifier, PPA+L, and PEGylated nanoamplifier+L. These groups were subjected to 24 h and 48 h MTT assays (Figure S14A-B). Simultaneously, B16 cells were also treated with the five groups for 12h, followed by co-cultivation with DC cells. The final results were consistent with the previous findings (Figure S14C-D).

### *In vivo* tumor targeting

**Figure 5:**
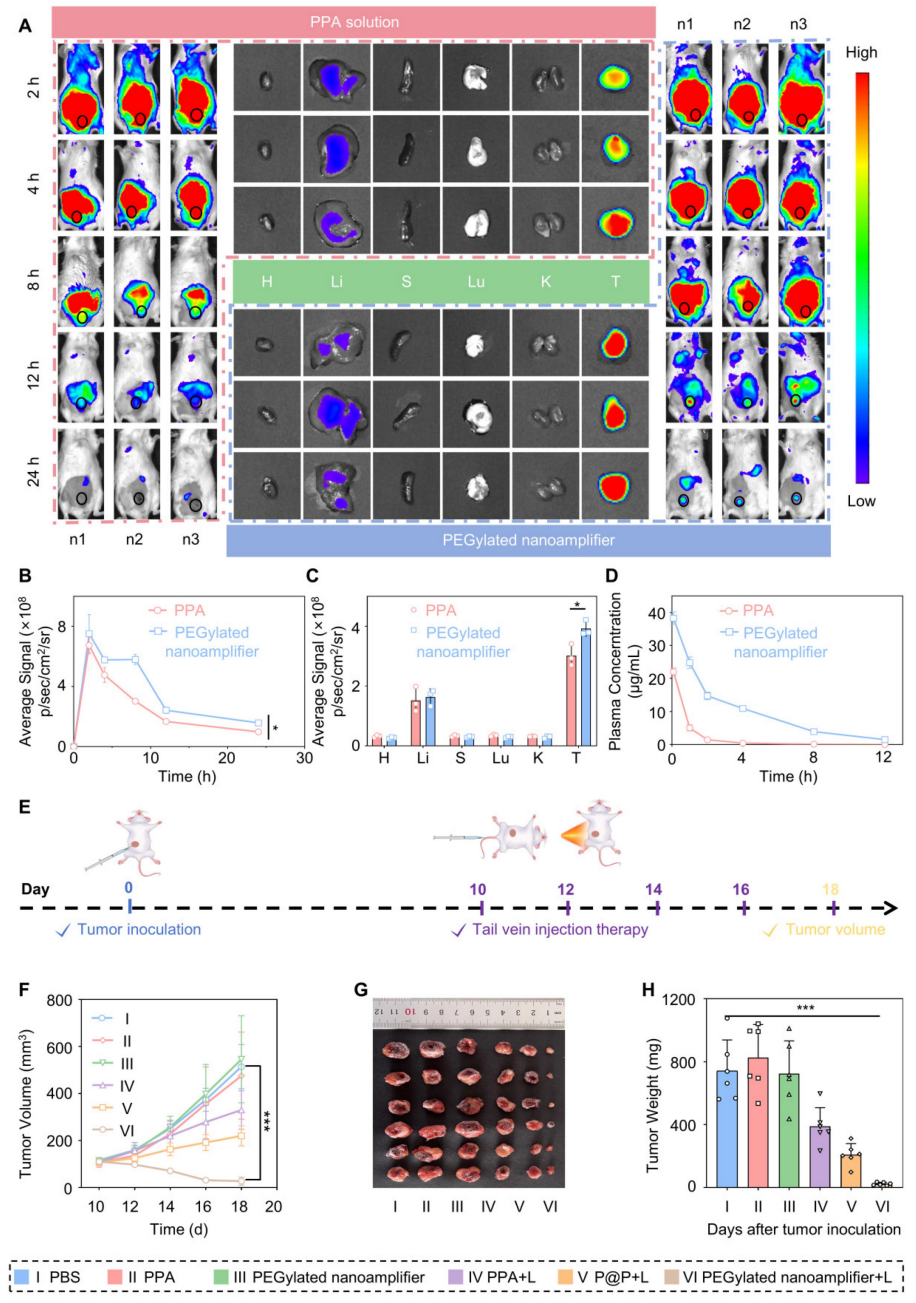
** Drug distribution *in vivo* and antitumor efficacy.** (A) Distribution of PPA in various organs and tumors in tumor-bearing mice at 8, 12, and 24 h after tail vein injection was observed using an *in vivo* imaging system. (B) Analysis of PPA fluorescence intensity in the tumor sites of mice at 8, 12, and 24 h (n = 3). (C) Analysis of PPA fluorescence intensity in various organs and tumors of tumor-bearing mice at 24 h (n = 3). (D) Pharmacokinetic analysis of PPA and PEGylated nanoamplifier in mice (n = 3). (E) Schematic diagram of the construction and treatment process for in situ 4T1 tumor-bearing mice. (F) Analysis of tumor growth curve data (n = 6). (G) Images of tumors from mice treated with different groups. (H) Analysis of tumor weight in mice treated with different groups (n = 6). PBS (I), PPA (II), PEGylated nanoamplifier (III), PPA+L (IV), P@P+L (V), PEGylated nanoamplifier+L (VI). *P*-value: ns (no significance) *p* > 0.05, *p < 0.05, **p < 0.01, ***p < 0.001.

Before performing the tail vein injection, we conducted a hemolysis test. The experiment was divided into three groups, including PEGylated nanoamplifier, positive control, and negative control groups. As shown in Figure S15A-B, at 0 h, there was no significant difference in the appearance of cell hemolysis between the three groups. Even after 3 h, there was no difference between the PEGylated nanoamplifier group and the negative control group, indicating that the reagent did not cause hemolysis at the concentration used for tail vein injection. We injected PEGylated nanoamplifier and PPA formulations via the tail vein and monitored the fluorescence intensity distribution of PPA within the body using an *in vivo* imaging system. Fluorescence images showed that at 2 h, there was no significant difference in fluorescence intensity at the tumor site between the PEGylated nanoamplifier and the PPA groups. Over time, from 2 to 24 h, the fluorescence intensity in the PPA group at the tumor site exhibited a rapid decline. In the PEGylated nanoamplifier group, the fluorescence intensity throughout the body decreased at 4 h and 8 h. However, the fluorescence at the tumor site remained relatively stable, which could be attributed to the accumulation of PEGylated nanoamplifiers at the tumor site. Overall, the PEGylated nanoamplifier group showed higher fluorescence intensity at the tumor site and throughout the body than the PPA group after 2 h. Notably, neither PEGylated nanoamplifier nor PPA formulations accumulated in the heart, spleen, lungs, and kidneys, but there was a small accumulation in the liver (Figure 5A-C). As shown in Figure 5D, pharmacokinetic analysis revealed that the fluorescence from the PPA formulation diminished rapidly. In contrast, there was a gradual decrease in the fluorescence from the PEGylated nanoamplifier, indicating improved *in vivo* circulation time of the PEGylated nanoamplifier following intravenous injection. These findings suggested that the PEGylated nanoamplifier had superior biodistribution and pharmacokinetics, providing significant advantages for tumor treatment.

### Tumor eradication and reshaping of the tumor microenvironment

Previous data indicated promising anticancer properties of the PEGylated nanoamplifier, including effective* in vitro* tumor killing and immune activation, along with excellent* in vitro* tumor targeting. Therefore, we conducted experiments to evaluate the *in vivo* efficacy of PEGylated nanoamplifier and its ability to remodel the immune microenvironment. We established an *in situ* tumor model in BALB/c mice. The mice were randomly assigned to six groups: PBS, PPA, PEGylated nanoamplifier, PPA+L, P@P+L, and PEGylated nanoamplifier+L. On day 0, 4T1 tumor cells were subcutaneously injected. On day 10, intravenous injections and light treatments were administered every two days, and the mice were euthanized on day 18 (Figure 5E). Compared to the PBS and PPA+L groups, the primary tumor volume and weight were significantly smaller in the PEGylated nanoamplifier+L-treated group, indicating its pronounced therapeutic effect *in vivo*, consistent with the *in vitro* experiments (Figure 5F-H). Furthermore, the body weight in the 6 groups of mice remained largely unchanged (Figure S16).

Mice were randomly selected from each group for biosafety evaluation and heart, liver, spleen, lungs, and kidneys were subjected to H&E staining. As shown in Figure S17, no significant tissue damage was observed. Additionally, the liver and kidney biochemical blood markers, including alanine aminotransferase (ALT), aspartate aminotransferase (AST), creatinine (CRE), and blood urea nitrogen (BUN), were assessed and found to be within normal ranges (Figure S18A-D).

**Figure 6:**
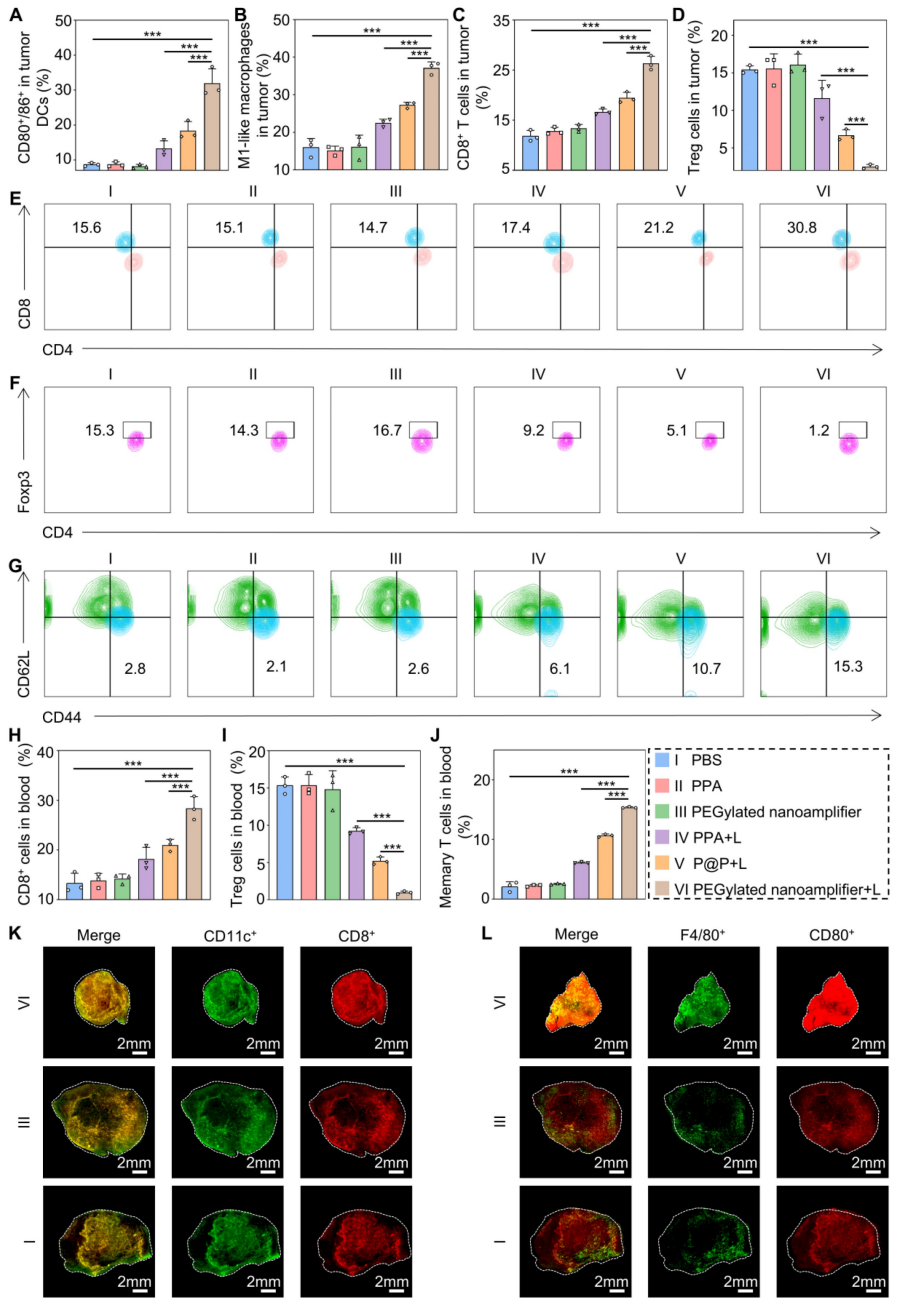
** Immune remodeling within the tumor and in the blood.** (A) Evaluation of CD80^+^/86^+^ cell statistics in tumors (n = 3). (B) Evaluation of M1-like macrophage statistics in tumors (n = 3). (C) Evaluation of CD8^+^ T cell statistics in tumors (n = 3). (D) Evaluation of Treg cell statistics in tumors (n = 3). (E) Evaluation of CD8^+^ T cells in blood using flow cytometry (n = 3). (F) Evaluation of Treg cells in blood using flow cytometry (n = 3). (G) Evaluation of Treg cells in Memory T cells in blood using flow cytometry (n = 3). (H) Evaluation of CD8^+^ T cells in blood using statistical analysis (n = 3). (I) Evaluation of Treg cells in blood using statistical analysis (n = 3). (J) Evaluation of Memory T cells in blood using statistical analysis (n = 3). (K) Immunofluorescence staining of tumors from different groups showing DCs detected with CD11c antibody (green) and CD8^+^ T cells detected with CD8 antibody (red). (L) Immunofluorescence staining of tumors from different groups, showing macrophages detected with F4/80 antibody (green) and CD80 antibody (red). PBS (I), PPA (II), PEGylated nanoamplifier (III), PPA+L (IV), P@P+L (V), PEGylated nanoamplifier+L (VI). *P*-value: ns (no significance) *p* > 0.05, *p < 0.05, **p < 0.01, ***p < 0.001.

Subsequently, we employed flow cytometry to quantify the immune cells in the blood and tumors of the mice. Figure 6A-C and Figure S19-21 illustrate that the levels of CD80^+^/86^+^ DCs, M1 macrophages, and CD8^+^ T cells in the tumors were markedly elevated in the PEGylated nanoamplifier+L group relative to the other groups. This increase is attributed to the activation of immune cells by the Vit K2 adjuvant through the TLR pathway in the PEGylated nanoamplifier. Concurrently, the levels of Treg cells were notably lower in this group, which was beneficial for tumor elimination (Figure 6D, Figure S22). Figure 6E-G reveals changes in immune cells in the blood of the mice. In the PEGylated nanoamplifier group, CD8^+^ T cells and Memory T cells significantly increased compared to the other five groups, while Treg cells significantly decreased (Figure 6H-J). Furthermore, the fluorescence intensity of CD11C^+^, CD8^+^, F4/80^+^ and CD80^+^ in the PEGylated nanoamplifier+L group was significantly higher than in the other two groups, consistent with our previous findings (Figure 6K-L). Overall, the PEGylated nanoamplifier could eliminate tumors and reshape the tumor microenvironment.

### Therapeutic efficacy of the formulation on distant breast cancer metastases

**Figure 7:**
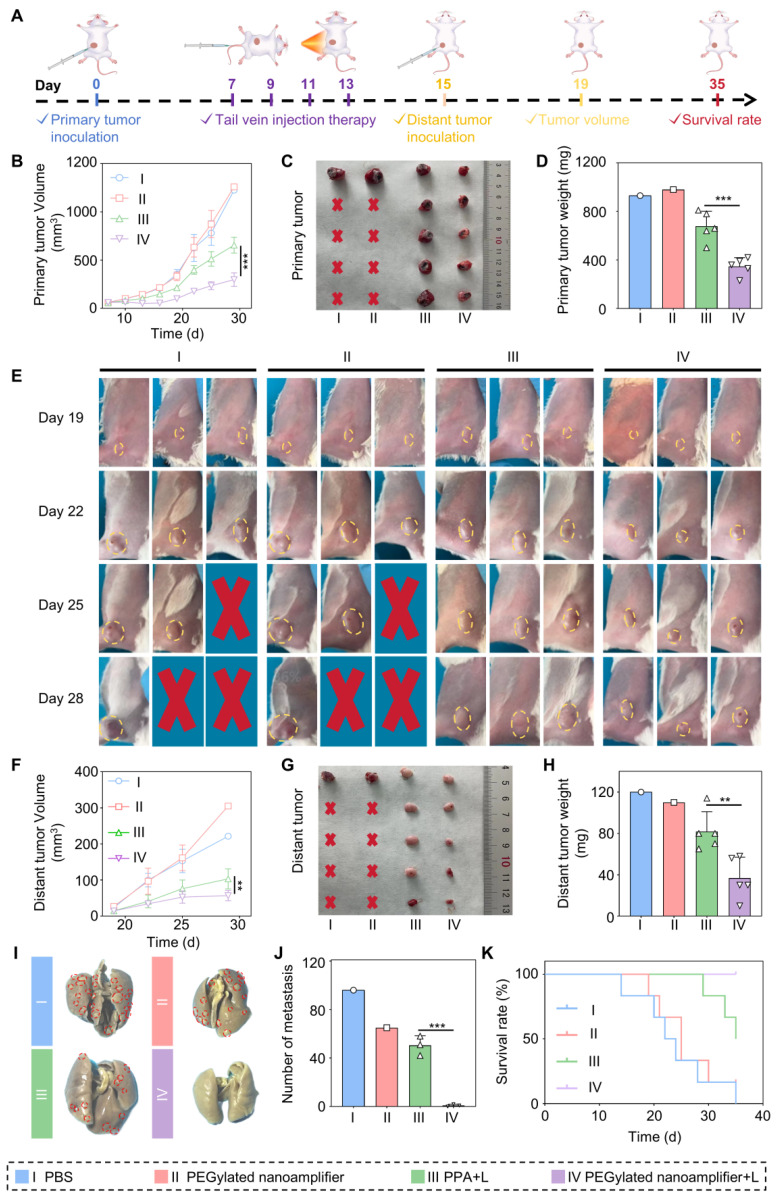
** Antitumor effects of different formulations in a breast cancer distant metastasis model** (A) Schematic diagram of the construction and treatment process in breast cancer distant metastasis model. (B) Analysis of the growth curve of the primary tumor data (n = 5). (C) Images of primary tumors from mice treated with different groups. (D) Analysis of primary tumor weight in mice treated with different groups (n = 5). (E) Images of tumor growth during treatment in different groups. (F) Analysis of growth curve of distant metastatic tumors data (n = 5). (G) Images of distant metastatic tumors from mice treated with different groups. (H) Analysis of distant metastatic tumor weight results in mice treated with different groups (n = 5). (I) Images of lung tumor metastasis. (J) Analysis of lung tumor metastasis (n = 3). (K) Survival curves of mice after treatment with different groups. PBS (I), PEGylated nanoamplifier (II), PPA+L (III), PEGylated nanoamplifier+L (IV). *P*-value: ns (no significance) *p* > 0.05, *p < 0.05, **p < 0.01, ***p < 0.001.

In the previous section, we validated the anti-tumor efficacy and immune microenvironment remodeling effects of PEGylated nanoamplifier on primary breast tumors. We established a breast cancer distant metastasis model to test the effectiveness of the nanoamplifier on metastatic breast cancer. Initially, mice were randomly divided into 4 groups. On day 0, primary tumors were implanted, followed by intravenous injections and light treatments on day 7, administered every 2 days for a total of 4 treatments. On day 15, tumors were again implanted in the hind legs of the mice, and tumor volumes were measured on day 19 (Figure 7A). In both primary and distant tumors, the treatment group exhibited significantly higher tumor suppression capability than the control group, with tumors treated with PEGylated nanoamplifier+L showing notably lower volume and weight than those treated with PPA+L (Figure 7B-H). These data provided evidence that PEGylated nanoamplifier induced immune memory in mice, resulting in significant anti-tumor effects upon the reccurence of the same tumor. The number of lung metastatic tumors was significantly fewer in the PEGylated nanoamplifier+L and PPA+L groups than in the other two groups (Figure 7I-J).

Due to mouse mortality during treatment, we further investigated mouse survival rates. The results showed that the survival rate in the PEGylated nanoamplifier+L treatment group was significantly higher than in the other three groups (Figure 7K). To further validate the efficacy of the PEGylated nanoamplifier+L group in combating distal tumors, mice were divided into PEGylated nanoamplifier+L and P@P+L, the treatment process is illustrated in Figure 7A. Following treatment, the volume and weight of proximal and distal tumors in the PEGylated nanoamplifier+L group were significantly smaller than those in the P@P+L group (Figure S23A-G). These findings indicated that PEGylated nanoamplifier significantly inhibited distant metastatic tumors in mice, reduced lung tumor metastasis, and prolonged mouse survival.

### Immune activation effects in human breast cancer organoid model

**Figure 8:**
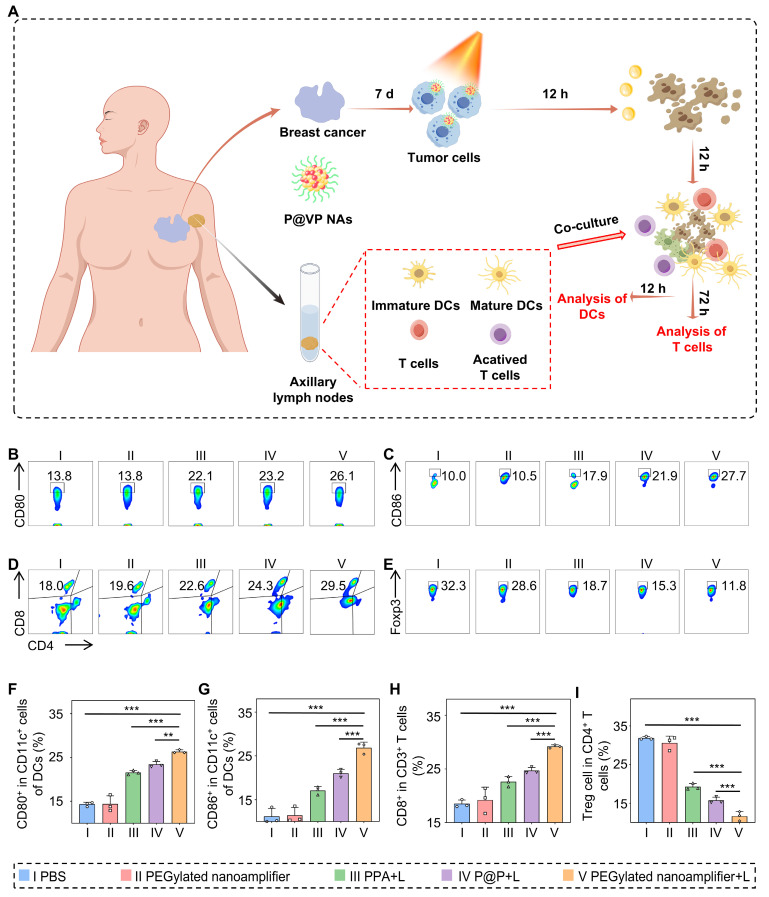
** Immune remodeling effects in patient-derived organoids model.** (A) Schematic diagram of immune remodeling in organoids. (B) Examination of CD80^+^ cells using flow cytometry (n = 3). (C) Examination of CD86^+^ cells using flow cytometry (n = 3). (D) Examination of CD8^+^ T cells using flow cytometry (n = 3). (E) Flow cytometry analysis of Treg cells (n = 3). (F) Data analysis of CD80^+^ cells (n = 3). (G) Statistical analysis of CD80^+^ cells (n = 3). (H) Statistical analysis of CD8^+^ T cells (n = 3). (I) Statistical analysis of Treg cells (n = 3). PBS (I), PEGylated nanoamplifier (II), PPA+L (III), P@P+L (IV), PEGylated nanoamplifier+L (V). *P*-value: ns (no significance) *p* > 0.05, *p < 0.05, **p < 0.01, ***p < 0.001.

We extracted organoids from fresh breast cancer tissues obtained from patients to further validate the exceptional immune activation capacity of the PEGylated nanoamplifier. The organoids were randomly divided into five groups: PBS (I), PEGylated nanoamplifier (II), PPA+L (III), P@P+L (IV), and PEGylated nanoamplifier+L (V). On day 7, the respective reagents were added to each group and laser exposure was applied as indicated. After 12 h, fresh lymphocyte-derived immune cells from the patients were introduced and co-cultured. The differentiation level of DCs was analyzed after 12 h using flow cytometry and the differentiation of T cells was assessed by flow cytometry after 72 h (Figure 8A). The activation level of DC cells in the PEGylated nanoamplifier+L group was markedly elevated compared to the other groups (Figures 8B-C, F-G). T cell stimulation in the PEGylated nanoamplifier+L group led to a notable rise in differentiated CD8^+^ T cells. In contrast, the differentiation of Treg cells significantly decreased, consistent with our previous experimental results (Figures 8D-E, H-I). Thus, the PEGylated nanoamplifier could effectively activate anti-tumor immunity in the human breast cancer tumor organoid model.

Despite the significant effects of our formulation, there are still some shortcomings. For example, it cannot completely eliminate the distant tumor, and while the photosensitizer exerts its cytotoxic effects, it inevitably damages immune cells. Future studies should address these issues.

## Conclusion

Previous studies have shown the potent tumor-killing effect of PDT. However, the tumor's immunosuppressive microenvironment allows cancer cells in its central region to evade detection by the body's immune cells. In this study, we explored the dual critical role of the nanoamplifier in directly killing tumor cells and remodeling the immune microenvironment. While the photosensitizer PPA kills tumor cells, computational simulation-screened adjuvant Vit K2 binds to TLRs on immune cells to remodel the immune microenvironment, activate immune cells, and eliminate distant metastatic cancer cells, preventing tumor recurrence. In summary, we have demonstrated the computer-assisted self-adjuvant treatment strategy as a potent therapeutic modality for cancer photoimmunotherapy.

## Supplementary Material

Supplementary figures.

Supplementary table.

## Figures and Tables

**Scheme 1:**
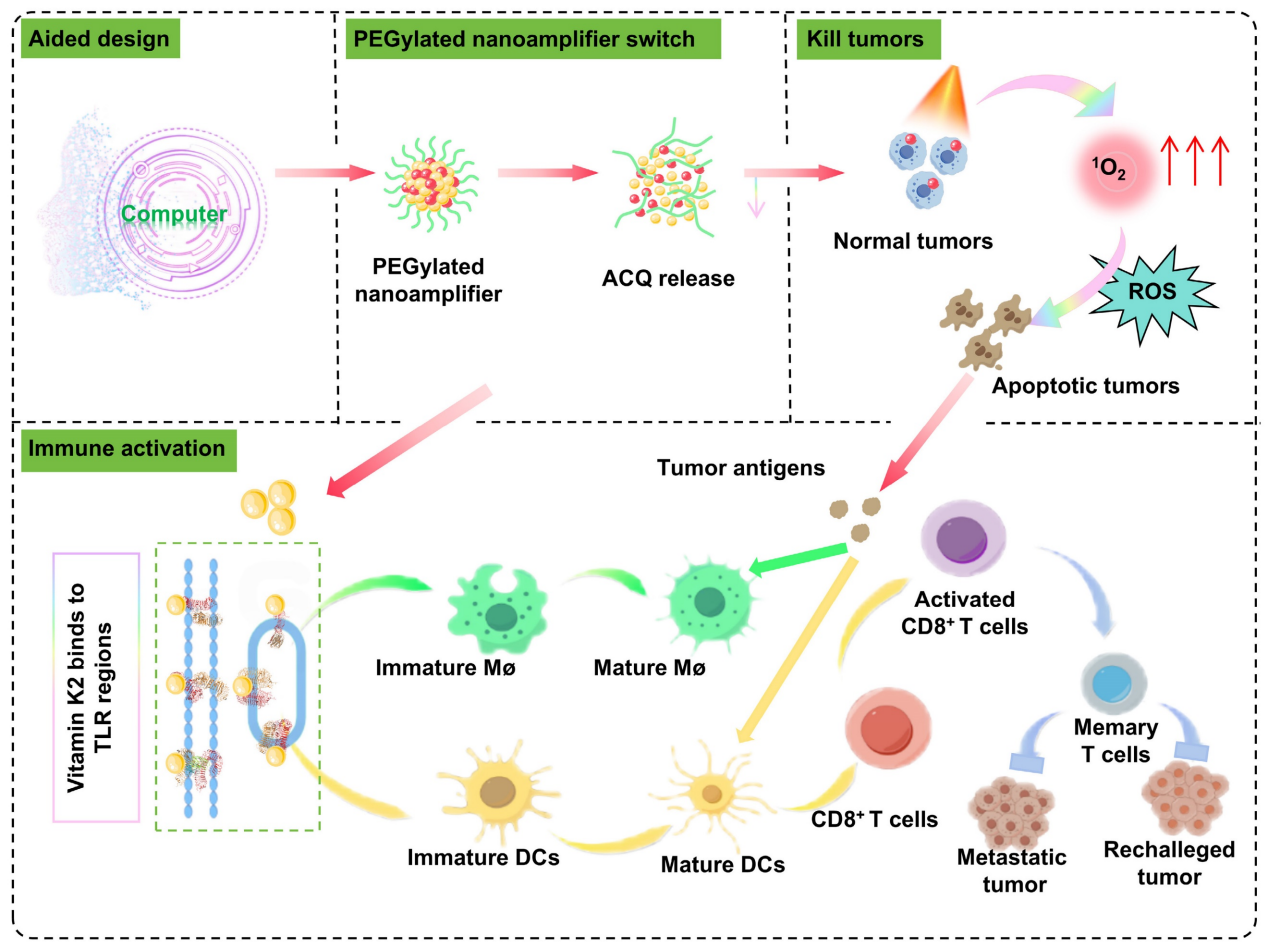
Mechanism diagram of the antitumor and immune activation effects of computer-aided designed self-adjuvanting nanoamplifier.
