# Supplementary material for: Molecular simulation-aided self-adjuvanting nanoamplifier for cancer photoimmunotherapy
Source: Theranostics. 2025 Jan 20;15(6):2451–69. doi: 10.7150/thno.102653 (PMC11840747; doi:10.7150/thno.102653)
Supplement: Supplementary file 1 — Supplementary figures. [file thnov15p2451s1.pdf]

Supplementary Information

Computer-aided design of vaccine combined with photosensitizer for synergistic anti-tumor therapy

Gu et al.

Supplement Information

Supplementary Table 2. The sequence of primers for DC 2.4 cell lines

mRNA	Forword primer（5'-3'）	Reverse primer（5'-3'）
**β-actin**	CTATTGGCAACGAGCGGTTC	ACTGTGTTGGCATAGAGGTCTT
**CCR7**	ACCCAGGAAAAACGTGCTGG	TTGAAGCACACCGACTCGTA
**IRF7**	CACCACACTACACCATCTACCT	ACAAGCACAAGCCGAGACT
**IP-10**	TGCCGTCATTTTCTGCCTCA	AGGCTCGCAGGGATGATTTC
**MIP-3β**	AGGCATAAATTGGAGCTGGTG	GCTCAGATGTCCTCCTGTGG
**IFN-β**	CGTGGGAGATGTCCTCAACT	CTGAAGATCTCTGCTCGGACC
**TNF-α**	CACCACGCTCTTCTGTCTAC	GGCTACAGGCTTGTCACTC
**IRF-3**	GCGTCTAGGCTGGTGGTTAT	GCCATGCTGTGTTTTGTCCC
**IL-10**	CCTCGTTTGTACCTCTCTCCG	AGGACACCATAGCAAAGGGC
**IL-18**	GGCTGCCATGTCAGAAGACT	GTCTGGTCTGGGGTTCACTG

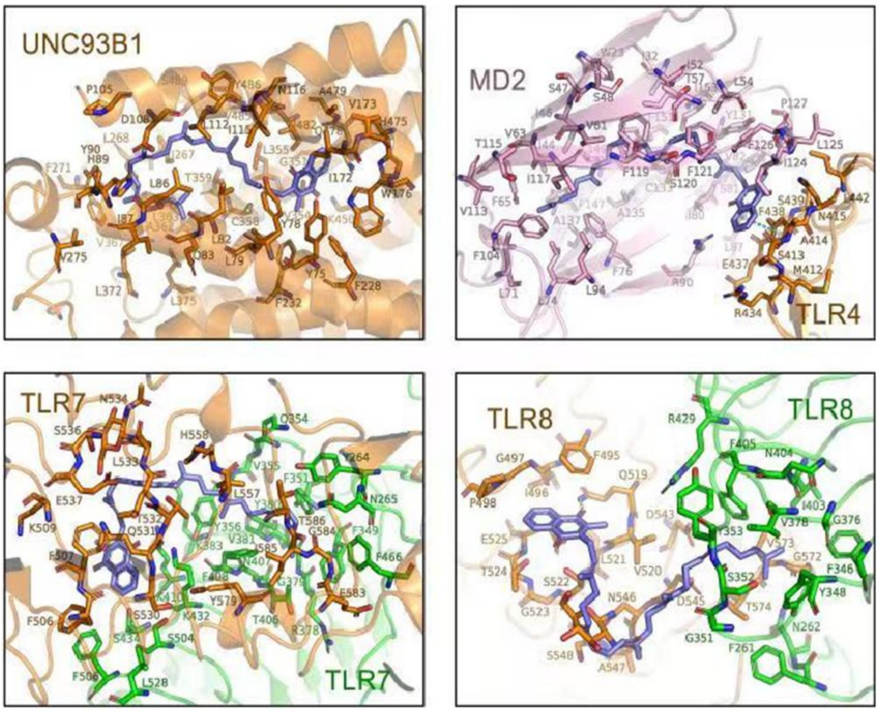

Supplementary Figure 1. A simulated illustration of Vit K2 docking with UNC93B1/TLR3, TLR4/MD2, TLR7, and TLR8.

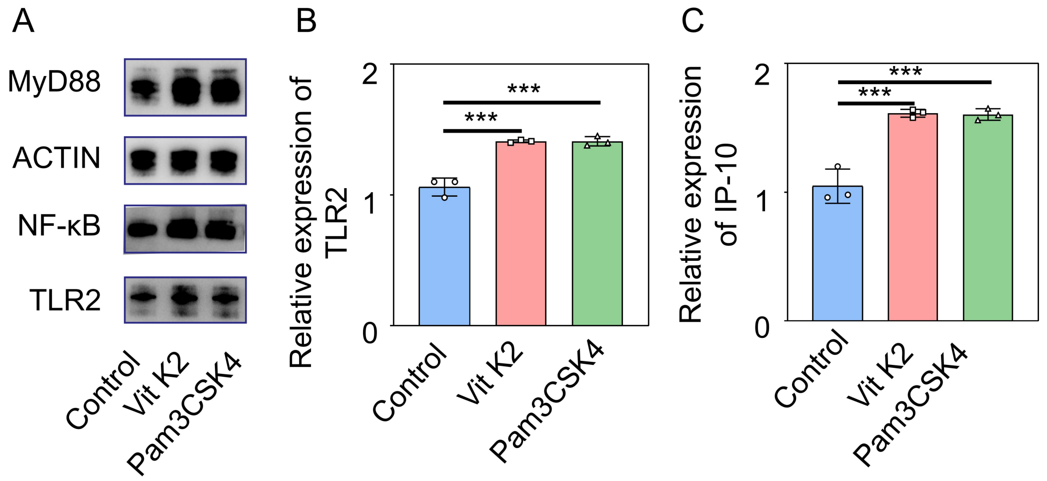

Supplementary Figure 2. (A) The expression levels of TLR2, MyD88, and NF-κB proteins in Control, Vit K2, or Pam3CSK4 group. (B-C) The relative RNA expression levels of TLR2 and IP-10 in Control, Vit K2, or Pam3CSK4 group (n = 3). P-value: ns (No Significance) $p > 0.05$, *$p < 0.05$, **$p < 0.01$, ***$p < 0.001$.

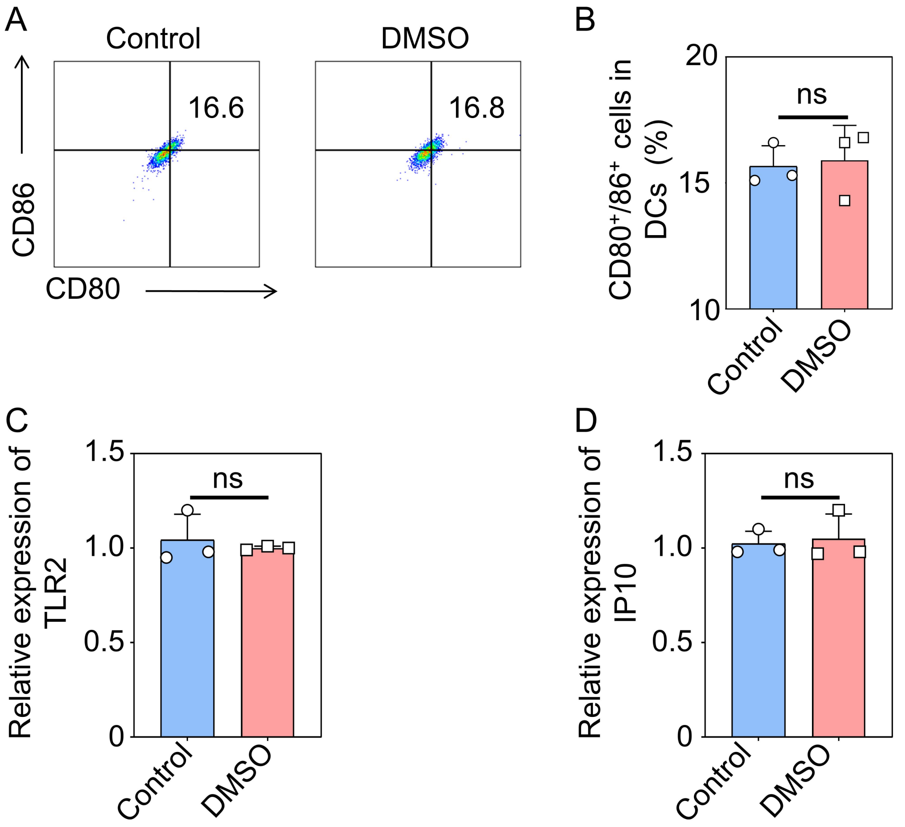

Supplementary Figure 3. (A) Examination of CD80+/86+ cells populations in DCs via flow cytometry following 12-hour exposure to Control and DMSO. (B) Statistical evaluation of CD80+/86+ cells in DCs (n = 3). (C-D) The relative RNA expression levels of TLR2 and IP-10 in Control and DMSO group (n = 3). P-value: ns (No Significance) $p > 0.05$, $*p < 0.05$, $**p < 0.01$, $***p < 0.001$.

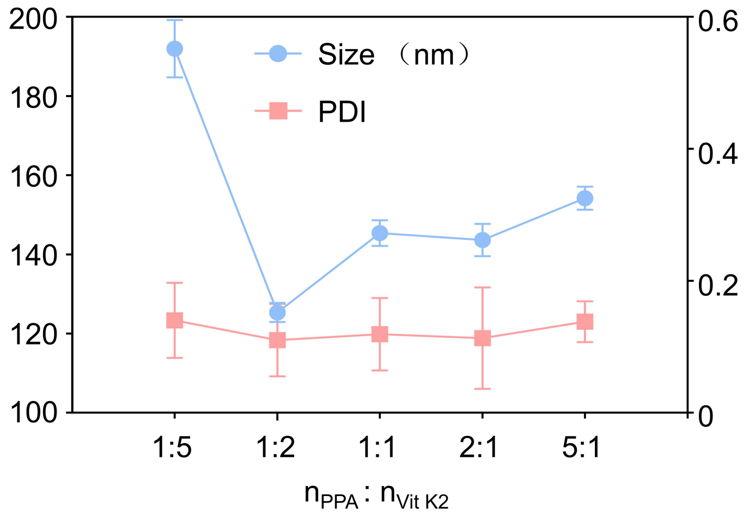

Supplementary Figure 4. Particle size and PDI of different molar ratios of PPA and Vit K2 (n = 3).

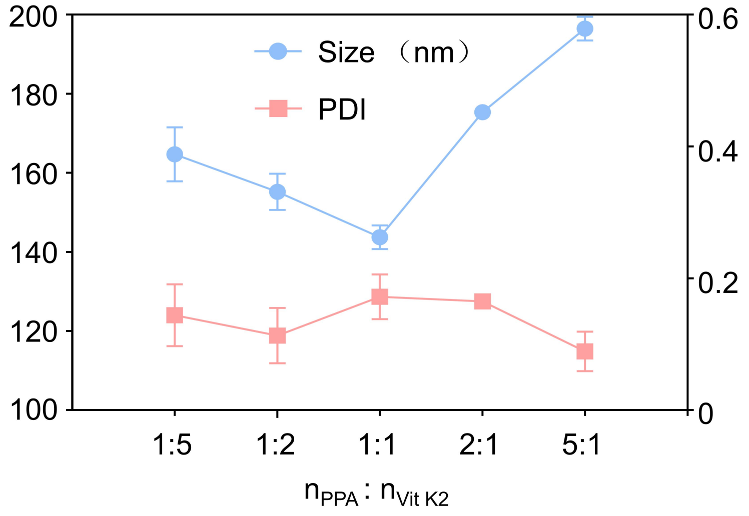

Supplementary Figure 5. Particle size and PDI of different molar ratios of PPA and Vit K2 with the addition of 15% DSPE-PEG2000 (n = 3).

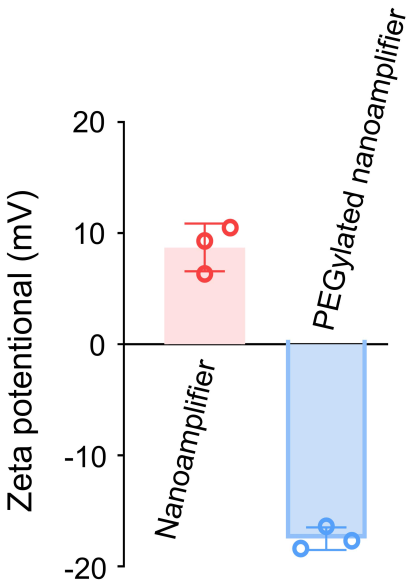

Supplementary Figure 6. Zeta potential of PEGylated nanoamplifier and nanoamplifier (n = 3).

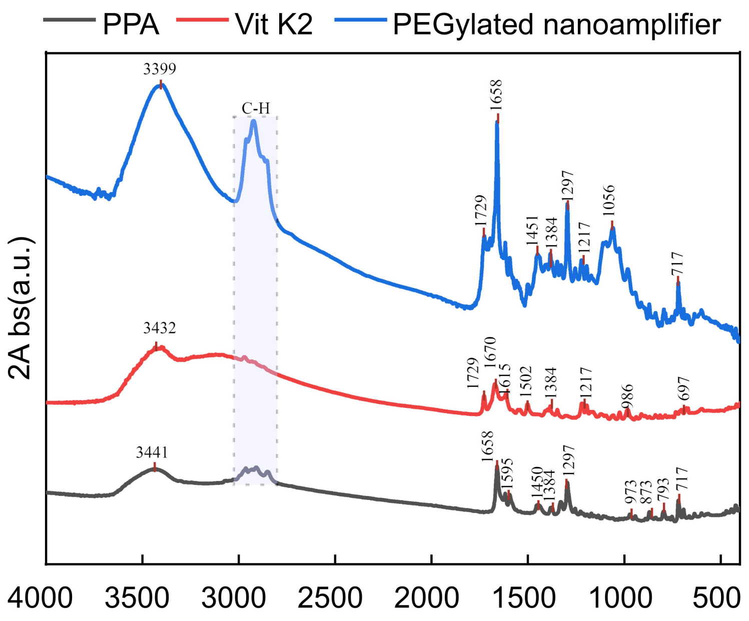

Supplementary Figure 7. FITR analysis of the PEGylated Nanoamplifier.

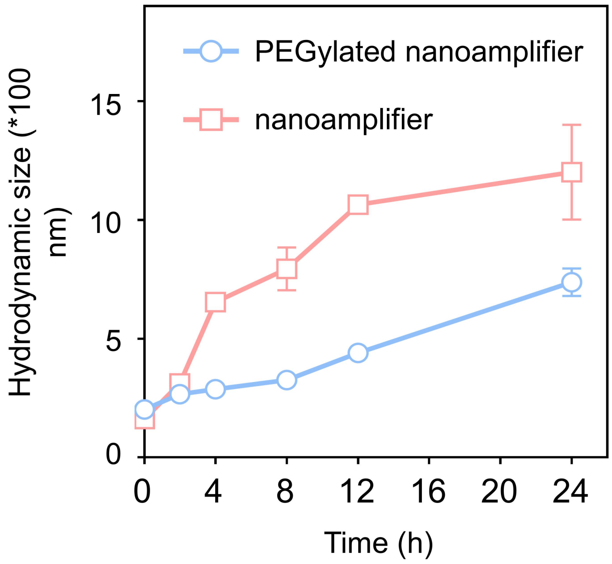

Supplementary Figure 8. Analysis of particle size of PEGylated nanoamplifier NAs in 10% FBS at time points 0h, 2h, 4h, 8h, 12 h, and 24h (n = 3).

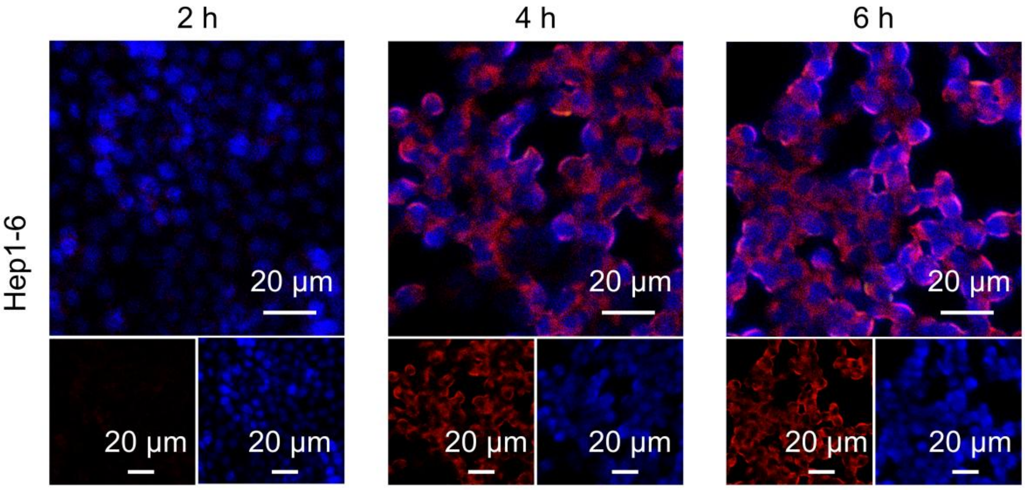

Supplementary Figure 9. Uptake of PEGylated nanoamplifier by Hep1-6 cells at 2, 4, and 6 hours.

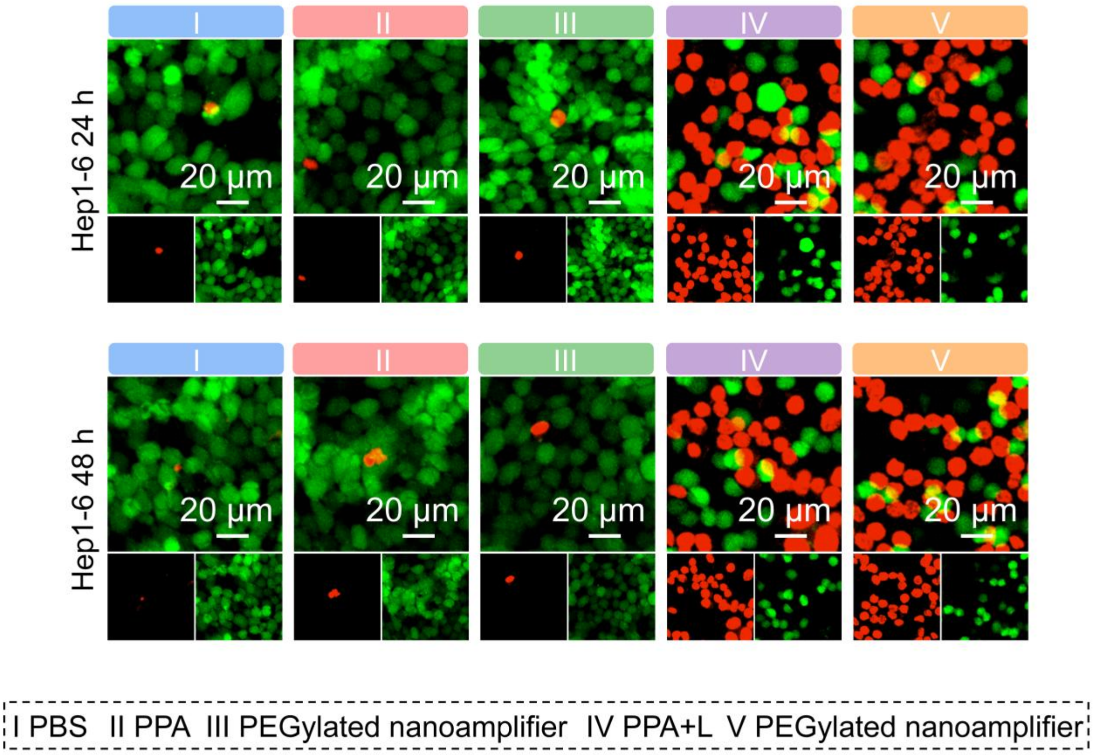

Supplementary Figure 10. Live/dead images of Hep1-6 cells under confocal microscopy (Green indicates healthy cells, while red represents apoptotic or necrotic cells).

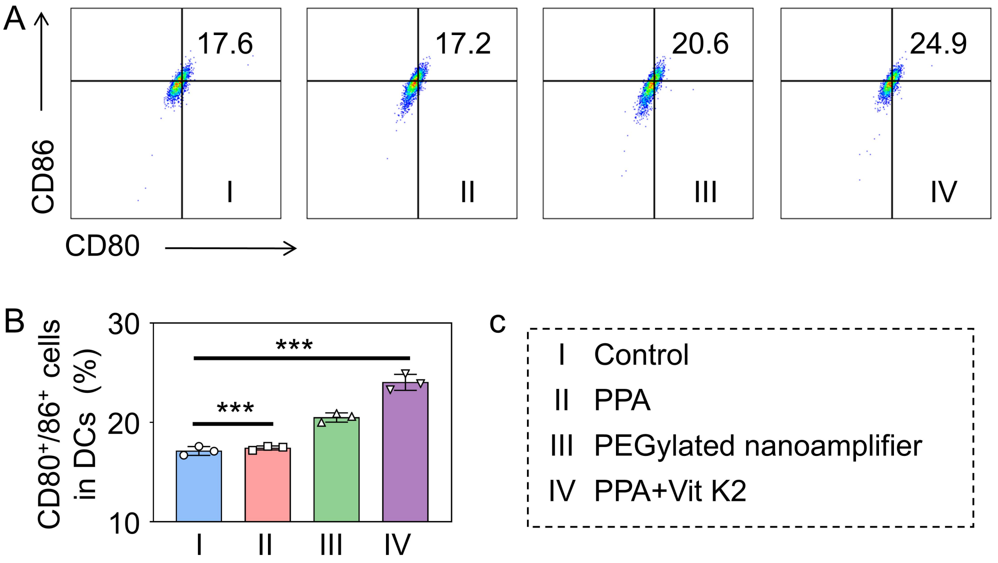

Supplementary Figure 11. (A) Examination of CD80+/86+ cells populations in DCs via flow cytometry following 12-hour exposure to Control, PPA, PEGylated nanoamplifier, and PPA+Vit K2. (B) Statistical evaluation of CD80+/86+ cells in DCs (n = 3). P-value: ns (No Significance) $p > 0.05$, *$p < 0.05$, **$p < 0.01$, ***$p < 0.001$.

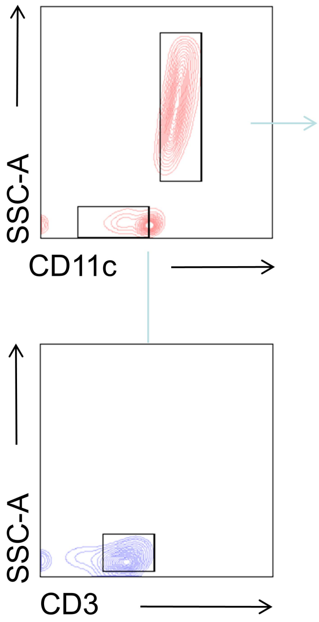

Supplementary Figure 12. Schematic representation of gating strategy for flow cytometry analysis (the axes are logarithmic).

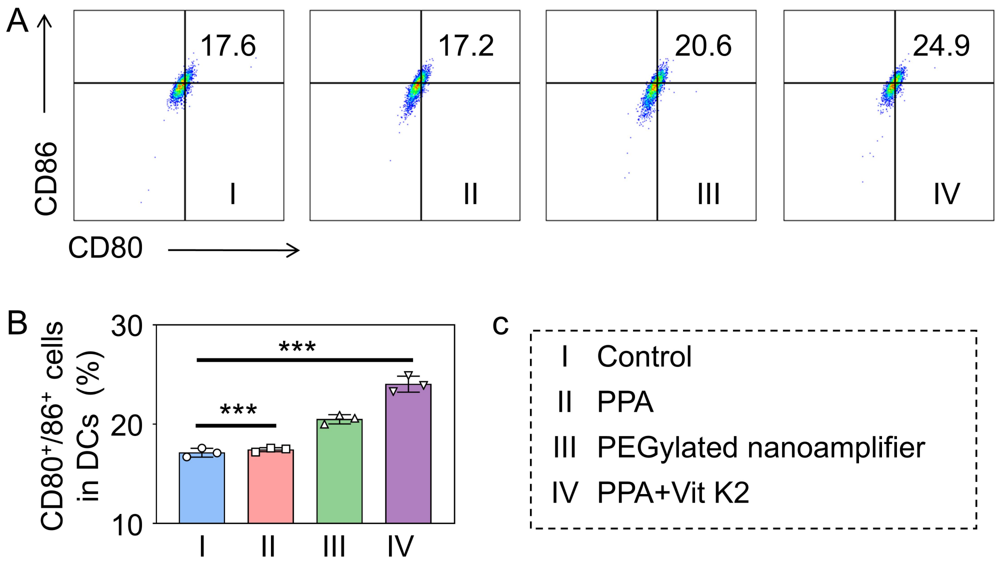

Supplementary Figure 13. (A) Evalution of CD3^{+} cells in CD11c^{-} using flow cytometry analysis (n = 3). (B) Evalution of CD3^{+} cells stastistics in CD11c^{-} (n = 3). P-value: ns (No Significance) $p > 0.05$, *$p < 0.05$, **$p < 0.01$, ***$p < 0.001$.

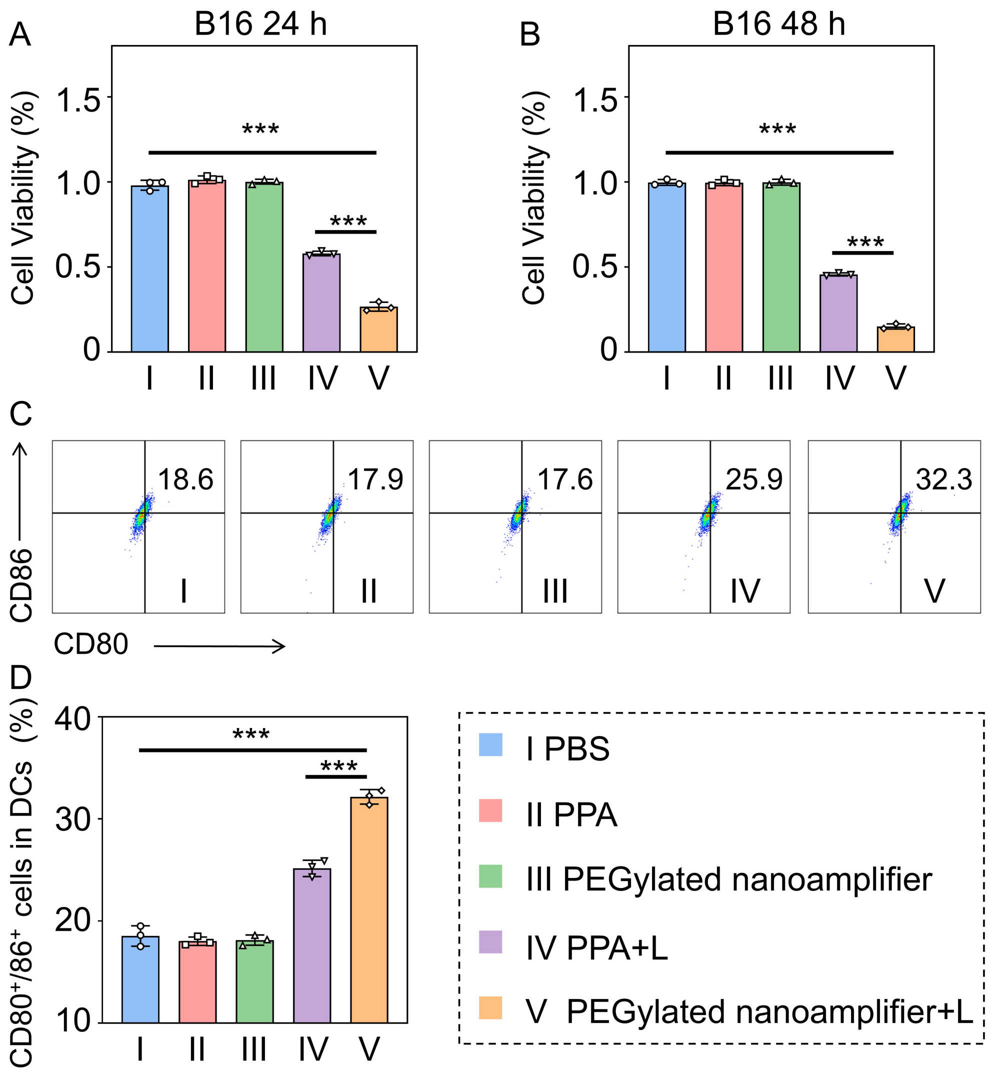

Supplementary Figure 14. (A-B) Cytotoxicity analysis of B16 cells after 24 or 48 h of treatment with PBS, PPA, PEGylated nanoamplifier, PPA+L or PEGylated nanoamplifier+L groups (n = 3). (C) Evalution of $CD80^+/86^+$ cells in DCs using flow cytometry analysis (n = 3). (D) Evalution of $CD80^+/86^+$ cells stastistics in DCs (n = 3).

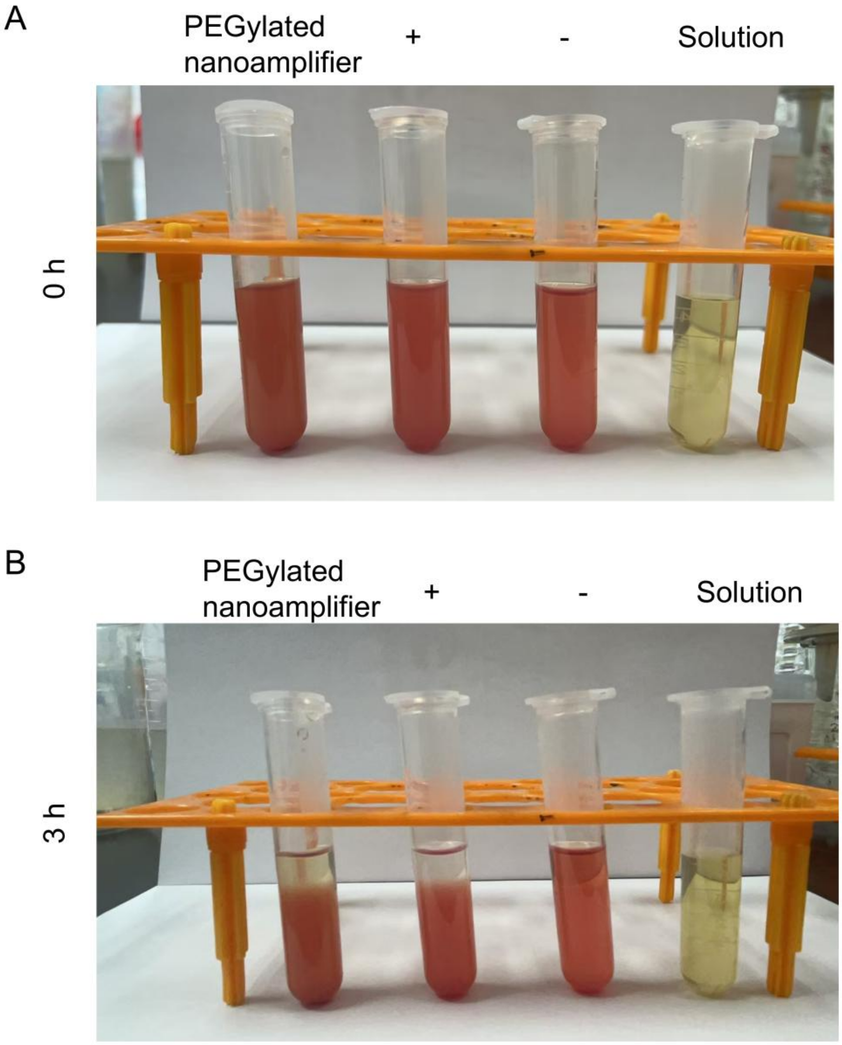

Supplementary Figure 15. (A) Photo showing test tubes containing red blood cells with different solutions and a test tube with PEGylated nanoamplifier solution at 0 h. (B) Photo showing test tubes containing red blood cells with different solutions and a test tube with PEGylated nanoamplifier solution at 3 h.

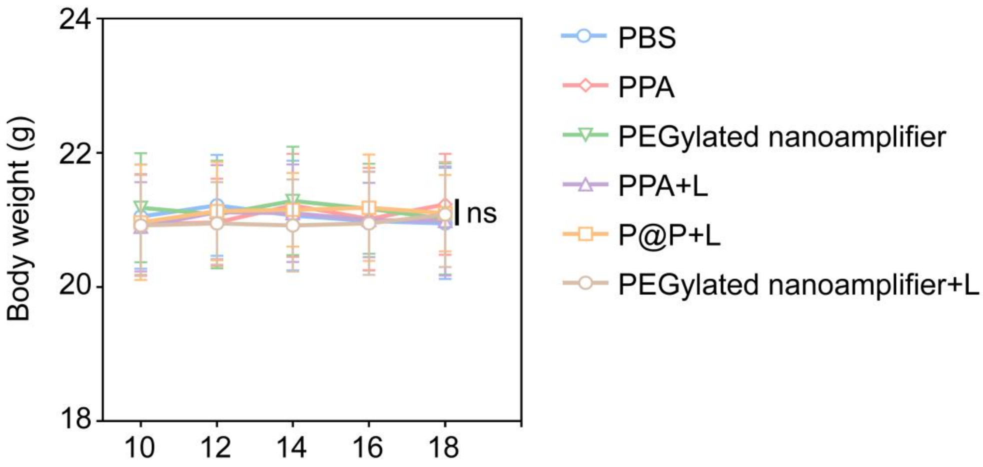

Supplementary Figure 16. Body weight changes in mice during treatment (n = 6). *P*-value: ns (No Significance) $p > 0.05$, *$p < 0.05$, **p < 0.01, ***p < 0.001.

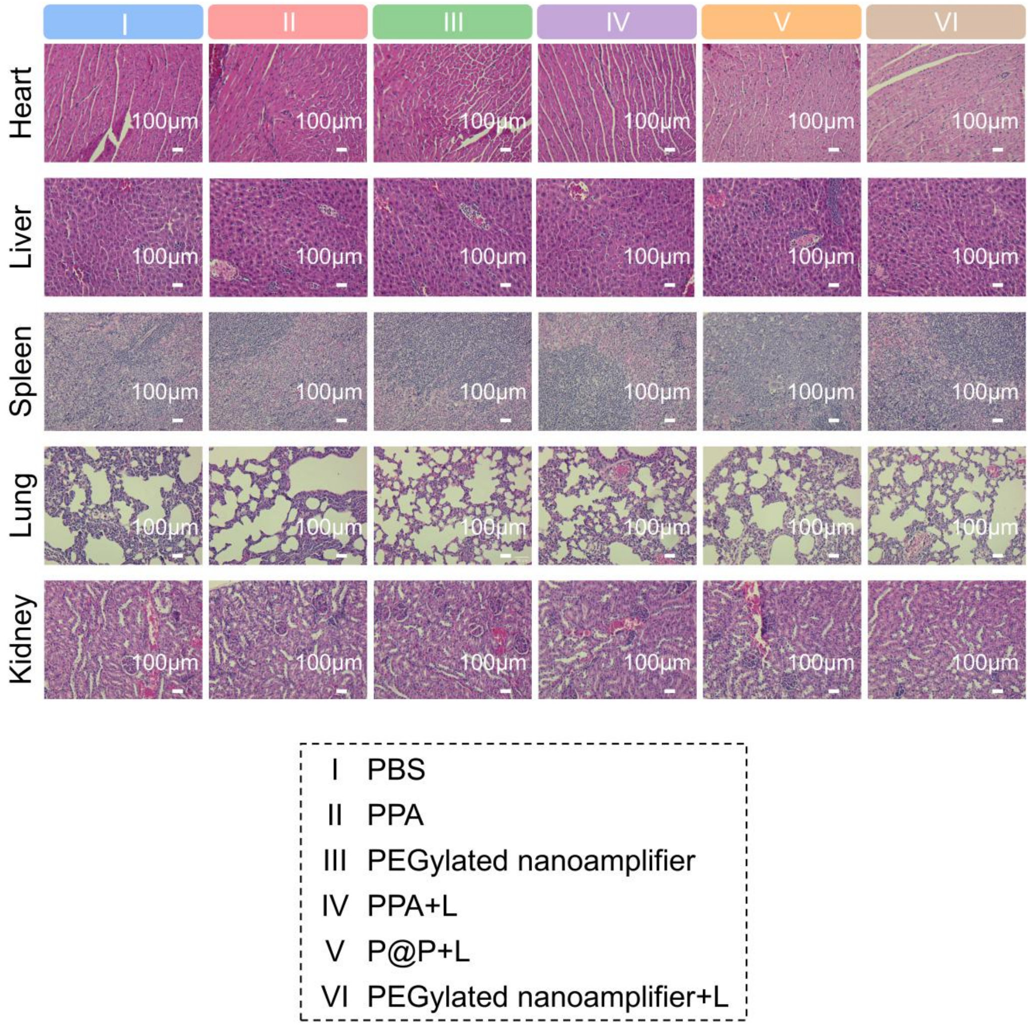

Supplementary Figure 17. Microscopic images of H&E-stained sections of the heart, liver, spleen, lungs, and kidneys from mice in different experimental groups.

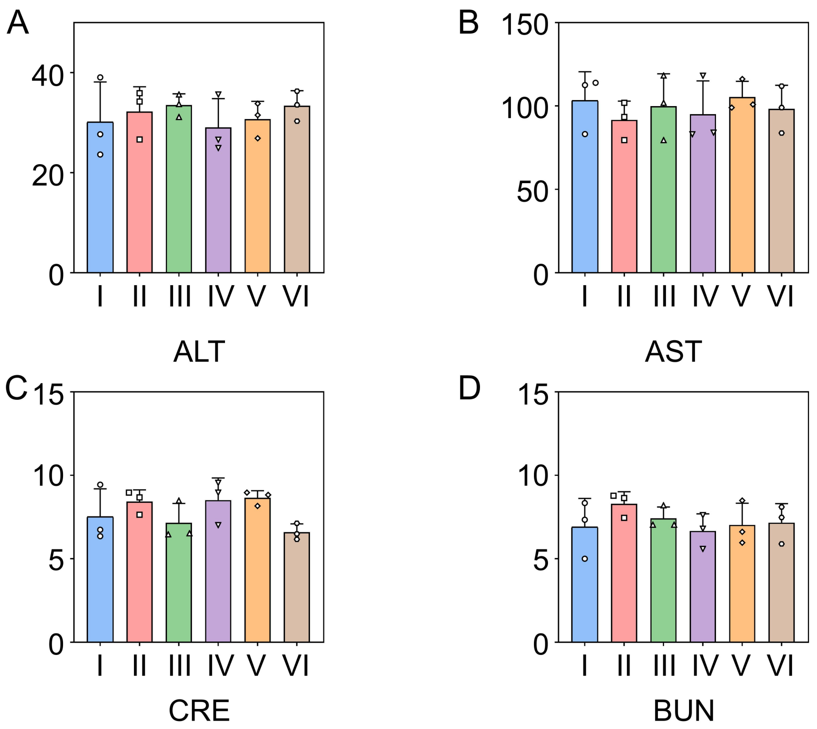

Supplementary Figure 18. (A) Statistical results of ALT levels in blood. (B) Statistical results of AST levels in blood. (C) Statistical results of CRE levels in blood. (D) Statistical results of BUN levels in blood.

.

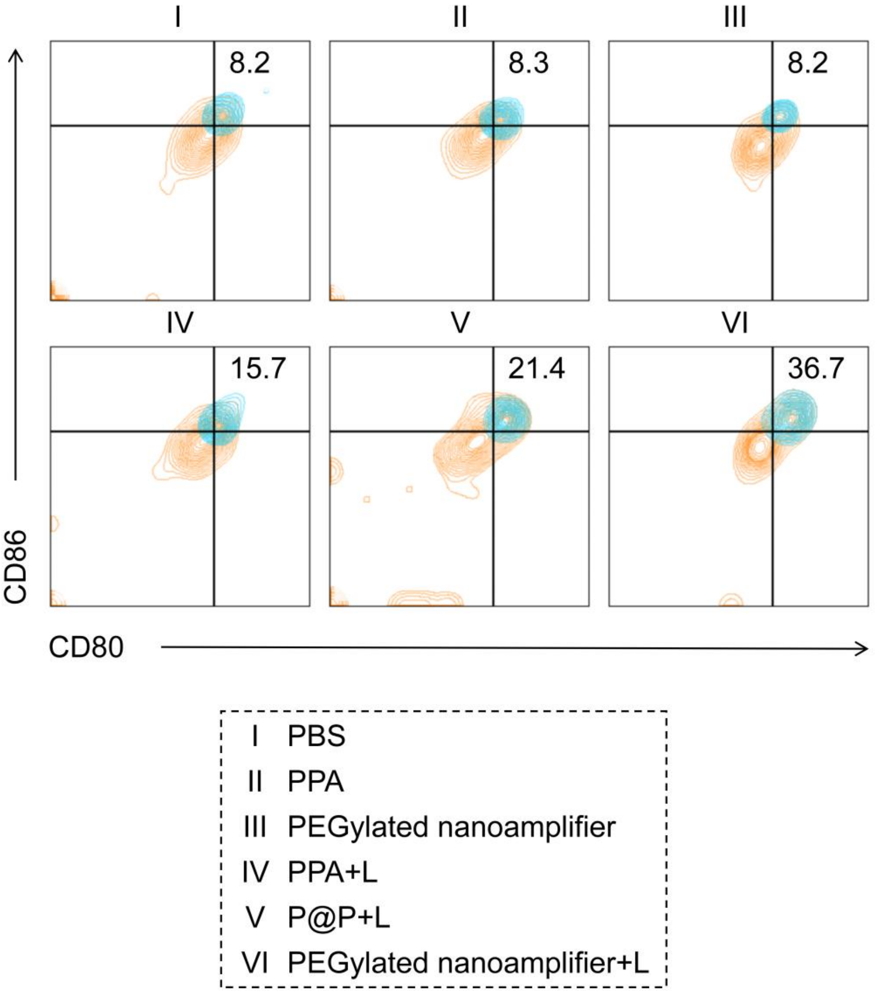

Supplementary Figure 19. Flow cytometry analysis of CD80$^+$/ CD86$^+$ cells (n = 3).

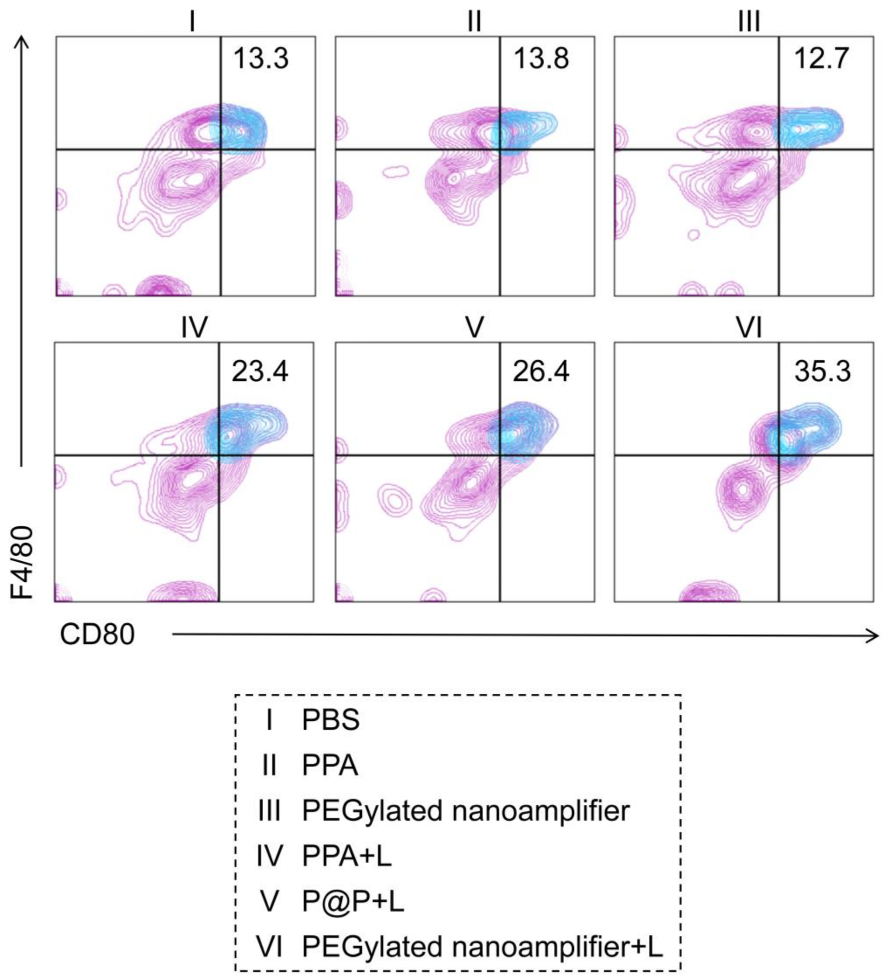

Supplementary Figure 20. Flow cytometry analysis of M1-like macrophage in tumor (n = 3).

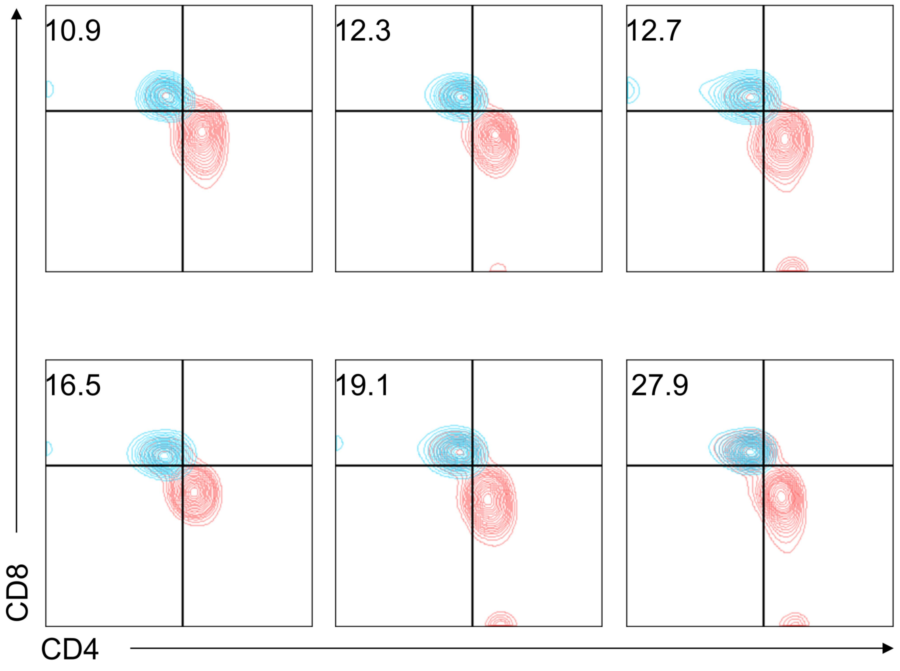

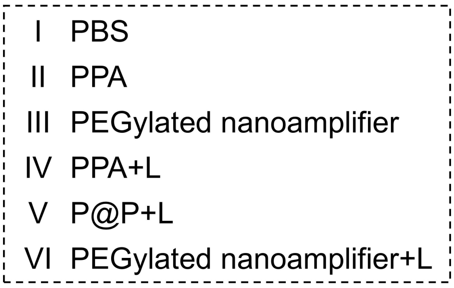

Supplementary Figure 21. Flow cytometry analysis of CD8+ T cells in tumor (n = 3).

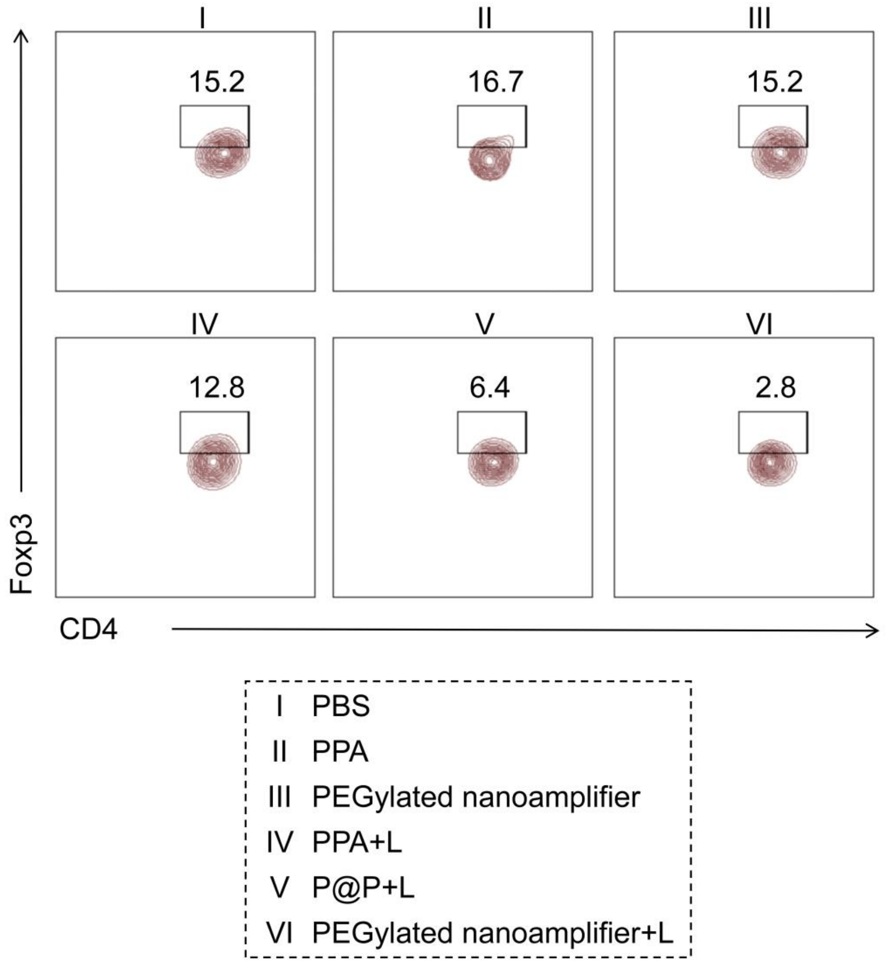

Supplementary Figure 22. Flow cytometry analysis of Treg cells in tumor (n = 3).

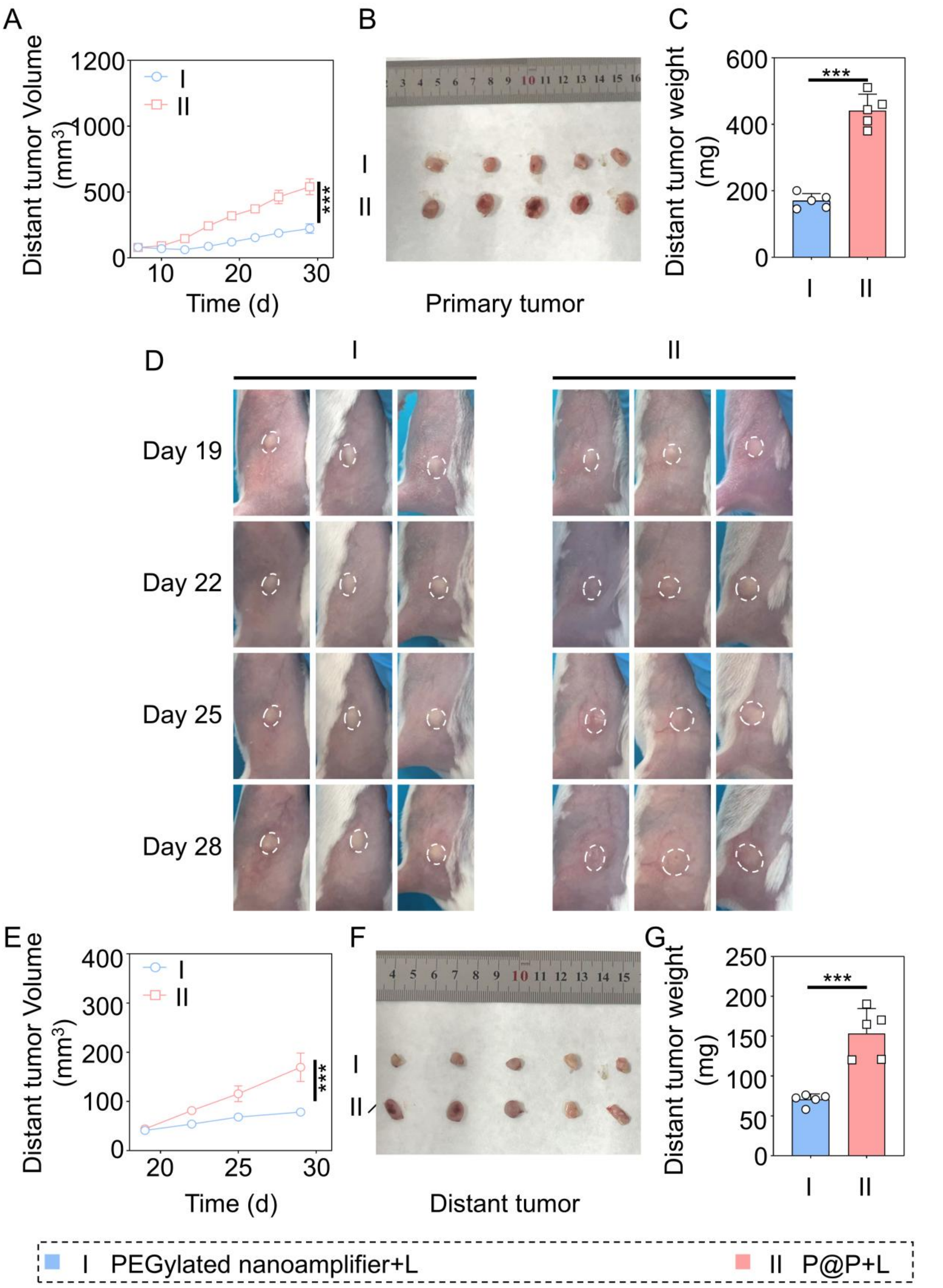

Supplementary Figure 23. Antitumor effects of different formulations in a breast cancer distant metastasis model. (A) Analysis of growth curve of the primary tumor data (n = 5). (B) Images of primary tumors from mice treated with different groups. (C) Analysis of primary tumor weight results in mice treated with different groups (n = 5). (D) Images of tumor growth during treatment in different groups. (E) Analysis of

growth curve of distant metastatic tumors data (n = 5). (F) Images of distant metastatic tumors from mice treated with different groups. (G) Analysis of distant metastatic tumor weight results in mice treated with different groups (n = 5). *P*-value: ns (No Significance) $p > 0.05$, $^{*}p < 0.05$, $^{**}p < 0.01$, $^{***}p < 0.001$.
